# Diagnostic, Prognostic, and Therapeutic Role for Angiogenesis Markers in Head and Neck Squamous Cell Carcinoma: A Narrative Review

**DOI:** 10.3390/ijms241310733

**Published:** 2023-06-27

**Authors:** Lara Alessandrini, Laura Astolfi, Antonio Daloiso, Marta Sbaraglia, Tiziana Mondello, Elisabetta Zanoletti, Leonardo Franz, Gino Marioni

**Affiliations:** 1 Surgical Pathology and Cytopathology Unit, Department of Medicine (DIMED), University of Padova, 35100 Padova, Italy; lara.alessandrini@aopd.veneto.it (L.A.); marta.sbaraglia@unipd.it (M.S.); 2 Bioacustic Research Laboratory, Department of Neuroscience (DNS), University of Padova, 35100 Padova, Italy; laura.astolfi@unipd.it; 3 Otolaryngology Section, Department of Neuroscience (DNS), University of Padova, 35100 Padova, Italy; antoniodaloiso96@gmail.com (A.D.); tiziana.mondello1995@gmail.com (T.M.); elisabetta.zanoletti@unipd.it (E.Z.); leonardo.franz@unipd.it (L.F.); 4 Phoniatrics and Audiology Unit, Department of Neuroscience (DNS), University of Padova, 31100 Treviso, Italy; 5 Artificial Intelligence in Medicine and Innovation in Clinical Research and Methodology (PhD Program), Department of Clinical and Experimental Sciences, University of Brescia, 25100 Brescia, Italy

**Keywords:** squamous cell carcinoma, head and neck, angiogenesis, hypoxia, endothelial, prognosis, target therapy

## Abstract

Despite refinements to diagnostic and therapeutic approaches over the last two decades, the outcome of patients with head and neck squamous cell carcinoma (HNSCC) has not shown substantial improvements, especially regarding those with advanced-stage disease. Angiogenesis is believed to be a turning point in the development of solid tumors, being a premise for mass growth and potential distant dissemination. Cancer-induced angiogenesis is a result of increased expression of angiogenic factors, decreased expression of anti-angiogenic factors, or a combination of both. The assessment of angiogenesis has also emerged as a potentially useful biological prognostic and predictive factor in HNSCC. The aim of this review is to assess the level of current knowledge on the neo-angiogenesis markers involved in the biology, behavior, and prognosis of HNSCC. A search (between 1 January 2012 and 10 October 2022) was run in PubMed, Scopus, and Web of Science electronic databases. After full-text screening and application of inclusion/exclusion criteria, 84 articles are included. The current knowledge and debate on angiogenesis in HNSCC presented in the eligible articles are stratified as follows: (i) diagnostic markers; (ii) prognostic markers; (iii) predictive markers; and (iv) markers with a potential therapeutic role. Angiogenesis is a biological and pathological indicator of malignancies progression and has negative implications in prognosis of some solid tumors; several signals capable of tripping the “angiogenic switch” have also been identified in HNSCC. Although several studies suggested that antiangiogenic agents might be a valuable adjunct to conventional chemo-radiation of HNSCC, their long-term therapeutic value remains uncertain. Further investigations are required on combinations of antiangiogenic agents with conventional chemotherapeutic ones, immunotherapeutic and molecularly targeted agents in HNSCC. Additional data are necessary to pinpoint which patients could benefit most from these treatments.

## 1. Introduction

Carcinomas that develop from mucosal epithelium of the upper aerodigestive tract rank as the sixth most common human cancer [1]. Head and neck squamous cell carcinomas (HNSCCs) may involve different sites including the sino-nasal district, the oral cavity, the oro-pharynx, the nasopharynx, the hypopharynx, and larynx. Tobacco, alcohol, occupational factors, and viruses—such as Human Papillomavirus (HPV)—are the main risk factors, which have a cumulative effect over time, resulting in an increased cancer incidence in the elderly (more than 70% of deaths from HNSCCs occur over the age of 70) [2,3]. The main factors related with recurrence risk and survival in patients with HNSCC are the direct invasion of adjacent tissues and regional lymph node metastasis [4,5]. Despite refinements to diagnostic and therapeutic approaches over the last two decades, mainly because of the relevant heterogeneity of these tumors, the outcome of patients with HNSCC has not shown substantial improvements, especially regarding those with advanced TNM stage disease, with a five-year overall survival (OS) rate around 50% [6].

Although the American Joint Committee on Cancer (AJCC) TNM staging system is routinely used in order to stratify patients and to have different risk groups, patients’ outcomes have long appeared to be heterogeneous within each stage, thus suggesting the existence of further prognostic factors that are not comprised in the traditional staging systems [7,8]. As a result, markers suitable to improve outcome discrimination are still needed, possibly reflecting biological features involved in determining tumor aggressiveness, which may also become targets for therapy.

From the primary site to the metastatic propagation, the tumor growth is led by the complex relationship between it and the microenvironmental system [9]. Increasing evidence regarding the impact of tumor microenvironment upon tumor cell biological behavior has accumulated in view of the complex interplay among endothelial, inflammatory, and immune cells, as well as extracellular matrix (ECM) signaling molecules [10]. The key role of the genesis of new blood vessels within the tumor microenvironment has long been regarded as fundamental in the progression of many solid tumors [11,12,13,14,15]. Angiogenesis is considered as a decisive step towards solid tumor growth, being a premise for proliferation, invasion, and migration [16]. Unbalanced expression of the pro- and anti-angiogenic factors from the tumor and surrounding tissue leads to tumor angiogenesis [17]. Over the past decade, analyzed by immunohistochemistry, the state of angiogenesis has become a prognostic and predictive biological factor in the staging in human solid malignancies [18]. Mean microvessel density (MVD) has been tested in many human malignancy histological types, resulting as a powerful, even if often independent, prognostic indicator [19,20,21]. In addition, a significant association between one of the main regulators of angiogenesis, vascular endothelial growth factor (VEGF) expression, and prognosis has been reported [22,23]. In many studies, tissue expression levels of angiogenic factors are related to the potential for tumor spread; therefore, they are considered as predictive indicators in identifying high-risk patients with poor prognoses [24,25,26]. At the same time, therapeutic approaches based on inhibiting neo-angiogenesis by either interfering with signal transduction pathways that regulate vessel genesis and growth or directly targeting tumor-associated endothelial cells have been proposed as promising strategies [27,28].

In order to verify the current level of knowledge on the markers of neo-angiogenesis in the biological field and the behavior and prognosis of HNSCC, a critical and narrative revision of the literature was carried out. The ultimate goal is to increase awareness of the role played by these biomarkers or pathways to clarify their fundamentals and stimulate the development of therapeutic objectives for medical treatments.

## 2. Materials and Methods

### 2.1. Protocol Registration

The review protocol of the present investigation was registered on PROSPERO, International prospective register of systematic reviews (Center for Reviews and Dissemination, University of York, York, UK) in October 2022 (registry number CRD42022367921).

### 2.2. Electronic Database Search

A literature review was conducted according to the Preferred Reporting Items for Systematic Reviews and Meta-Analyses recommendations [29]. The literature search for articles published between 1 January 2012 and 10 October 2022 was run in the following electronic databases: Pubmed, Scopus, Web of Science, and Cochrane. A combination of MeSH terms and free-text words were utilized to search for “head and neck”; “squamous cell”; “carcinoma”; and “angiogenesis” (Appendix A). The reference lists of all the included articles were accurately screened to identify other pertinent studies. References were exported to a Zotero bibliography manager (v6.0.10, Center for History and New Media, George Mason University, Fairfax, Virginia). After the duplicates were removed, the two reviewers (A.D. and T.M.) independently screened all titles and abstracts of the research and then evaluated the full texts of the eligible articles based on the inclusion criteria. Any disagreement between the reviewers involved in the literature search was resolved through discussion with all authors to reach a consensus.

### 2.3. Inclusion and Exclusion Criteria

Studies were deemed eligible when the following inclusion criteria were met: (i) confirmed pathological diagnosis of squamous cell carcinoma (SCC); and (ii) tissue specimen analysis performed through immunohistochemistry (IHC) or molecular methods (such as Western blot, enzyme-linked immunosorbent assay, quantitative real time protein chain reaction, etc.; for more information see Tables S1 and S2). Exclusion criteria were as follows: (i) preclinical (in vitro or non-human) models; (ii) retrospective series with less than 50 cases; (iii) lack of relevant data; (iv) non-original studies (i.e., reviews, recommendations, letters, editorials, and book chapters); and (v) non-English language reports. The papers were thoroughly screened for duplicates; only case-based studies without consistent overlap in the patients considered by others from the same research groups were analyzed.

### 2.4. Data Extraction and Quality Assessment

The authors analyzed the data from the available literature. The included studies were analyzed to extract available data and ensure eligibility for all patients. The risk of bias was considered for all included studies. Any disagreements about inclusion/exclusion of investigations were solved by a discussion among the study team members. The quality rating of each study was categorized as poor, fair, or good, according to the National Institutes of Health quality assessment tool for Observational Cohorts and Cross-Sectional Studies [30].

## 3. Results

### 3.1. Search Results and Quality Assessment

A total of 4119 titles were collected from the bibliographic research. After the duplicates were removed and exclusion of 2255 records due to coherence with the inclusion/exclusion criteria, 208 articles relevant to the topic were accurately evaluated. Two records were unavailable for retrieval; thus, 206 articles were assessed for eligibility, and 84 were included in the review [19,26,31,32,33,34,35,36,37,38,39,40,41,42,43,44,45,46,47,48,49,50,51,52,53,54,55,56,57,58,59,60,61,62,63,64,65,66,67,68,69,70,71,72,73,74,75,76,77,78,79,80,81,82,83,84,85,86,87,88,89,90,91,92,93,94,95,96,97,98,99,100,101,102,103,104,105,106,107,108,109,110,111,112].

According to the National Institutes of Health quality assessment tool for Observational Cohorts and Cross-Sectional Studies [30], 33 studies were deemed of Good quality (39.2%), 45 were Fair (53.6%), and 6 (7.1%) were found as Poor, due to the lack in reporting information on the series’ characteristics (see Tables S1–S3).

### 3.2. Features of the Studies

All the 84 studies included in the qualitative analysis were ex vivo tissue investigations based on histopathological analysis of tumor biopsies or surgical specimens [19,26,31,32,33,34,35,36,37,38,39,40,41,42,43,44,45,46,47,48,49,50,51,52,53,54,55,56,57,58,59,60,61,62,63,64,65,66,67,68,69,70,71,72,73,74,75,76,77,78,79,80,81,82,83,84,85,86,87,88,89,90,91,92,93,94,95,96,97,98,99,100,101,102,103,104,105,106,107,108,109,110,111,112]. Studies were published from 2012 to 2022. The median number of patients per study was 80 (range 50–941).

## 4. Discussion

Endothelial cells are responsible for triggering the formation of new blood vessels during tumorigenesis; these cells constitute the innermost layer of blood vessels. Diffusion is responsible for O_2_ supply and CO_2_ removal during the initial tumorigenesis; on the other hand, the growth requires increased supply of O_2_ as well as removal of metabolic waste [16].

**Figure 1:**
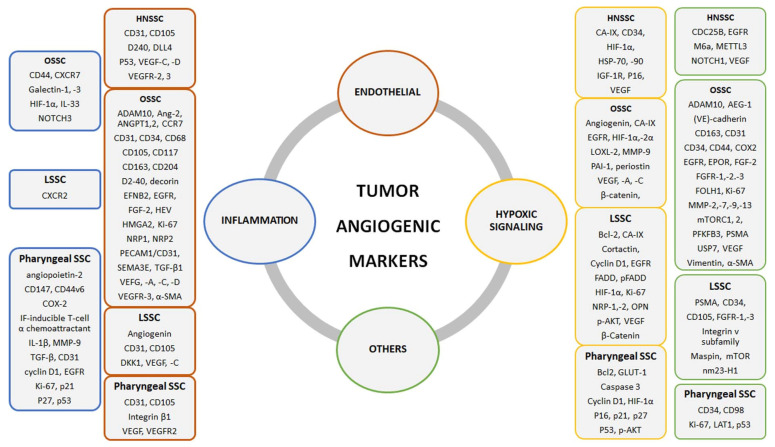
Markers involved in HNSCC and related to angiogenesis.

Figure 1 summarizes the molecular angiogenesis markers related to HNSCC, reported according to the main pathway activated and tumor site involved.

The current knowledge and debate on angiogenesis in HNSCC presented in the eligible articles was stratified as follows: (i) Diagnostics and staging; (ii) Cancer risk assessment and prognosis (grouped by site whenever possible); (iii) Early detection and screening. Predictive markers; and (iv) Treatment and monitoring. Markers with potential therapeutic role.

### 4.1. Diagnostis and Staging (See Table S1)

Epidermal growth factor receptor (EGFR) has been found to be overexpressed on the cell surface of several types of tumors, including HNSCC. As a result, it represents one of the most established makers to identify and label cancer cells for diagnostic and experimental purposes. Although EGFR is not defined as an angiogenetic factor, it was investigated in HNSCC since it is active in various intracellular signal pathways, where it has been found involved in stimulating the angiogenesis in addition to cell proliferation, causing an inhibition of programmed cell death and a stimulation of progression in the cell cycle [113]. The role of EGFR upregulation in tumor development and progression through the mechanism of tumor angiogenesis has been demonstrated in several tumors, for instance non-small-cell lung cancer, head and neck, pancreatic, colorectal, breast, and brain cancers [113,114,115]. EGFR-based signaling has been also related with the VEGF biological pathway [109]. In HNSCC, the association of EGFR with angiogenesis was related to the activation of the signal-transducer and activator of transcription 3 (STAT3), thus inducing the transcription of VEGF [116]. This biological pathway represents another possible target for therapy, related to angiogenesis mechanisms.

Fibroblast Growth Factor Receptors (FGFRs) are a subgroup of tyrosine kinase receptors involved in several biological activities. The role of FGFRs in HNSCC behavior have been extensively studied [117,118]. The FGF–FGFR pathway has been reported as one of crucial actors on tumorogenesis. FGF-2 has been shown to promote tumor progression by increasing the expression of proteolytic enzymes and by paracrine-inducing the growth of vascular endothelial cells [119]. FGF-2 levels were significantly expressed in up to 60.38% of oral SCCs (OSCCs) [105]. In addition, the combined expression of FGF-2 and FGFR-2 was observed in the progression from a precancerous lesion into a malignant one [105]. According to the previous evidence, the group of Mariz et al. [103] reported similar results analyzing oral epithelial dysplasia (OEL) and OSCC. Elevated levels of FGF-2 and FGFR-1 were observed in high-grade lesions, namely OEL, rather than in low-grade lesions. Moreover, FGF-2 expression was related with malignant transformation of OEL to SCC [103]. Yang et al. [111] reported that the expression of ubiquitin-specific protease 7 (USP7), a member of the ubiquitin proteasome system, in OSCC tissues were mostly upregulated compared to surrounding healthy tissues by IHC analyses. As a result of these data, it can be argued that these indicators should be feasibly added to the histopathological evaluation of epithelial dysplasia routinely performed for the evaluation of progression and malignant transformation in PMOLs.

Fibroblasts, together with immune cells, pericytes, and endothelial cells, contribute to the formation of the tumor microenvironment suitable for the proliferation of cancer, invasion, angiogenesis, metastases, and chemo-resistance [120].

In OSCC, the expression of neurogenic locus notch homolog protein 3 (NOTCH3) plays an essentially role in angiogenesis regulation, promoting cell–cell contact between fibroblasts and cancer cells. The expression of NOTCH3 by cancer-associated fibroblasts has a paracrine effect in cancer cells; for this reason, it was suggested as a marker of poor prognosis in OSCC patients [35]. In contrast, NOTCH1 has a tumor-suppressor role in the pathogenesis of OSCC [35]. 

Galectins are proteins that are able to perform many functions in multiple cellular processes, including inflammation and tumor development, playing a role in dysregulation of cell cycle, apoptosis, adhesion and cell migration, and angiogenesis. Aggarwal et al. [31] enrolled 60 randomly selected OSCC patients and divided them into two groups: ‘early’, at stages I and II tumors, and ‘late’, at stages III and IV. Thirty age- and gender-matched healthy subjects were involved as the control group. In OSCC patients, the expressions of galectin-1 and galectin-3 were detected to be significantly higher than in controls, both in blood and in tumor tissues. By logistic regression analysis, they measured a risk that was about three times higher in the OSCC subjects, with an overexpression of these proteins [31]. Aggarwal et al. [31] assumed that the two isoforms played a role in tumor growth both by interacting at the immune level and by inducing angiogenesis. In general, galectins compromise the balance of the production of cytokines T helper 1 (Th1) and Th2, with a resulting immunosuppressive effect. In particular, galectin-1 is known to be an enhancer of interleukin-10 (IL-10) cell expansion and of CD25+ Forkhead box P3+ (Foxp3) T Regulatory (Treg) cells’ immunosuppressive activity [121,122]. In a later study, Tokmak et al. [37] confirmed the correlation between galectin-3 expression and tumor grade and invasion by immunohistochemical analysis in OSCC. Regarding the angiogenetic effect in OSCC patients, a direct correlation between VEGF and galectin-1/galectin-3 expression was identified [31]. Aggarwal et al. [31] pointed to this finding by the fact that it has been shown in the literature that the binding of galectin-1 to Neuropilin-1 (NrP1) on endothelial cells can facilitate the BrP1/VEGFR-2 mediated signaling pathway by improving the phosphorylation of VEGFR-2, leading to the activation of mitogen-activated protein kinases (MAPK), stress-activated protein kinase/c-Jun N-terminal kinase (SAPK/JNK) [123].

The extracellular matrix metalloproteinase inductor (EMMPRIN, also known as CD147) has been reported as an additional factor involved in tumor invasion and metastasis processes. In hypopharyngeal squamous carcinoma, CD147 appears to mediate extracellular matrix (ECM) degradation by stimulating matrix-metalloproteinases (MMPs) synthesis and promoting angiogenesis by stimulating VEGF expression [39]. In SCC, the CD44v6, an isoform of CD44, reported a positive correlation with the development of lymph node metastasis, although the clinical significance remains controversial [39]. It was found that its metastatic action also acted as proangiogenic along with the over-expression of cyclooxygenase-2 (COX-2) and VEGF. COX2, in particular, is an enzyme that acts for the biosynthesis of prostaglandins. Its activity is to stimulate downstream to several inflammatory cytokines and oncogenes that lead to the development of inflammation and tumor growth [39].

VEGF and its receptors (VEGFR-1, -2 and -3) are the main actors of the signaling system that acts to regulate the processes of proliferation and migration of endothelial cells [124]. VEGF is the most known and potent angiogenic protein. Firstly, it is able to increase vascular permeability [113]. VEGF production is a consequence of the expression of cytokines, the activation of oncogenes and the suppression of antitumor genes [125]. VEGF is overexpressed in most human malignancies where endothelial cell proliferation, migration, and survival has been detected. Moreover, in several studies, it has been reported that VEGF overexpression correlates with a higher intra-tumor MVD [126]. Amongst five different types (VEGF-A, -B, -C, -D, and -F), VEGF-A and B have been reported as angiogenic factors, while VEGF-C showed a lymphangiogenic action [40]. Fei et al. [127] ranked 85 cases of tonsil SCC as low and high VEGF expression; in high expressers, the probability of detecting a CD31-rated MVD above 15 for High Power Field was significantly higher than in low expressers. Aggarwal et al. [40] reported that serum VEGF levels were significantly higher in OSCC patients, and that these expressions were directly correlated with the clinical stage evolvement and neck lymph node involvement. VEGF binds three types of receptors differing in signaling features of tyrosine kinase receptors: VEGFR1 (also known as flt-1), VEGFR2 (also known as Flk-1/KDR), and VEGFR3. Xu et al. [68] detected VEGFR2 both in endothelial cells and cancer cells in HNSCCs. Uzun et al. [67] evaluated the interplay between HPV and VEGFR2 expressions in oropharyngeal SCC. It is noteworthy that VEGFR2 was detected to be upregulated in carcinoma cells on HPV-negative carcinomas, while on the HPV-positive ones, it was detected to be upregulated in tumor-supporting blood vessels. In the latter patients, these associations resulted as indicators of poor prognoses. This preliminary evidence suggests the significance of deregulated VEGF signaling as prognostic marker for oropharyngeal SCC patients.

### 4.2. Cancer Risk Assessment and Prognosis (See Table S2)

#### 4.2.1. HNSCC

Hypoxia is a physiological phenomenon encountered in rapidly growing solid cancers, due to quantitative and qualitative alterations in tumor vessels, leading to local reduction of oxygen availability [128]. Cancer cells respond to hypoxia in the microenvironment through transcription of many genes, including “hypoxia-inducible factor-1α” (HIF-1α), heat-shock proteins (HSPs), and angiogenesis-related molecules, such as VEGF [129]. HIF-1α is a heterodimeric transcriptional complex that acts as the main regulator of systemic and cellular oxygen homeostasis. Under hypoxic conditions, HIF-1α is activated and induces the transcription of over 60 genes in an effort to overcome hypoxia-mediated cell death. Among HIF1-regulated genes, there are angiogenic and proliferating factors, anaerobic glycolytic enzymes, glucose transporters, and others, which are involved in tumor growth and survival, invasion, angiogenesis, metastasis, and chemo-resistance [128]. HIF-1α overexpression has been disclosed to be related to a poor prognosis in many cancers [130]. In OSCCs, the reliability of HIF-1α as a prognostic indicator is debated because of possibly conflicting outcomes [131]. While Fillies and colleagues [131] found that over a population of 85 surgically treated T1-2 OSCCs described an association between HIF-1α overexpression and a favorable outcome in terms of improved survival, Zhou et al. [132], considering 1474 OSCC cases, found that the same marker was associated with advanced clinical stage, presence of neck lymph node metastases, and a worse prognosis. Regarding studies focused on HNSCCs in general, the vast majority of reports showed an association between high HIF-1α expression and poor prognosis and/or failure of treatment options [71,72,73,74,75,76,77,78,79,80,81,82,83,84,85,86,87,88]. A comprehensive investigation by Choi et al. [74] on 90 HNSCCs confirmed the relationship between HIF-1α and poor disease-free survival (DFS), despite the absence of distinction by site during data analysis. Swartz et al., instead, studied whether there were SCC site-related differences in HIF-1α expression and its effect on outcome in a total of 941 HNSCC patients [87]. In this study, the immunohistochemical expression of HIF-1α in tissue samples was significantly higher in OSCCs than in other sites, but high expression led to a better prognosis in OSCC in terms of both OS and DFS.

HSPs are a family of proteins categorized according to their molecular weights and are also known as ‘chaperones’. They are part of the process of protein folding in response to different cellular stresses, including hypoxic conditions. Besides preventing HIF-1α degradation, HSPs have important roles in oncogenesis and malignant progression and can also be used as targets for cancer treatment [133]. In their series of 90 HNSCCs, Choi and colleagues [74] found that HSP70 overexpression, more frequently present in oro-pharyngeal SCC or HPV-positive cases, was associated with neck nodes metastasis occurrence and poor DFS. As HSP70 seems to determine anti-viral adaptive immune responses in various sites, it has been hypothesized that cellular immunity against human papilloma virus (HPV) might trigger HSP70 overexpression.

Insulin-like growth factor 1 receptor (IGF-1R) is also linked to a HIF-1-dependent signaling pathway. In HNSCC, IGF-1R activation leads to cell proliferation, HIF-1α ex-pression, and VEGF secretion. Choi et al. [74] disclosed a strong IGF-R1 overexpression only in HPV-positive SCCs. 

Another factor upregulated and activated by hypoxic conditions is carbonic an-hydrase IX (CA-IX). CA-IX is a member of the carbonic anhydrase family, which comprises transmembrane enzymes catalyzing the reversible hydration of carbon dioxide to carbonic acid. It acts in pH regulation by making the acidification of the microenvironment possible, enhancing cell growth and migration [134]. The meaning of CA-IX in HNSCC prognosis is debated. Considering tumor tissue from 100 patients with known HPV status and locally advanced HNSCC treated with concurrent chemo-radiotherapy (three-weekly cisplatin) or bio-radiotherapy (weekly cetuximab), Ou et al. [83] evaluated the expression level of CA-IX and the MVD determined as the density of CD34+ vascular structures. The investigation found a significant association between MVD and Union for International Cancer Control (UICC) stage and T classification, between CA-IX and UICC stage and N classification. Furthermore, a significant negative correlation between MVD and CA-IX expression was disclosed. Multivariable analysis highlighted that low MVD together with high CA-IX expression was an independent prognostic factor for worse loco-regional control in the whole population of HNSCCs but not in the p16+ sub-group.

A possible mechanism counterbalancing the hypoxia-induced biological cascade is related to the expression of the non-metastatic gene 23 (nm23) family. Such genes are re-sponsible for encoding several proteins that contribute to regulation of several biological processes, such as differentiation, proliferation, apoptosis, and molecular transportation. A total of 10 genes, encoded from nm23-H1 to nm23-H10, have been found in humans to date [135]. Nm23-H1 was the first metastasis suppressor to be discovered, and it has been the most studied because of its negative correlation with the onset, progression, and metastasis of several malignancies [136]. Nm23-H1 has long been regarded as a positive prognostic factor, protecting from nodal and distant metastases even in cases in which angiogenesis markers appear to be expressed [137,138]. Among different HNSCC types, the nm-23 biological effect has been characterized especially in laryngeal SCC (LSCC) [91,112].

Another molecule involved in cell cycle control and in the relationship with hypoxia-driven stimuli is mammary serine protease inhibitor (maspin). It is a tumor suppressor and is potentially prognostically relevant for HNSCCs, whose expression results in regulation of various biological processes. It is related to cell migration’s inhibition, cell invasion, and angiogenesis, as well in as apoptosis induction [139]. As a result of such biological properties, maspin expression in cancer cells has been regarded as a potential epiphenomenon of tumor-suppression mechanisms [140]. Within the HNSCC field, the biological and prognostic value of maspin have been studied in particular regarding LSCCs, and it has been reported to vary depending on the subcellular localization of its expression [91,141,142].

Downstream the hypoxia-driven processes and crucial effectors of biological pathways are the MMPs, which form a family of 28 zinc-dependent endo-peptidases divided into several sub-families according to their substrate specificities [143]. All MMP family members share a catalytic domain and a pro-peptide domain [144] and are considered primary contributors to ECM degradation in tumor cell invasion [145]. Many MMPs are known to take part in the angiogenesis process. In such a context, their primary action is to degrade ECM components, leading together to the release of ECM-bound angiogenic factors, and enabling tumor stroma’s invasion by endothelial cells. This process results in new blood vessel formation [146]. Accumulating evidence has shown that MMPs are expressed in HNSCCs, playing a significant prognostic role. Such molecules and their biological interaction have been studied and characterized from a prognostic point of view, especially in the OSCC setting.

CD31 is a member of the immunoglobulin-superfamily platelet endothelial cell adhesion molecule-1, which is highly expressed on endothelial cells’ surface and is involved in several tumors’ angiogenesis processes [147]. CD31 is a widely used marker to evaluate MVD. In 70 HNSCC cases, de Oliveira et al. [46] reported that patients with loco-regional metastasis presented a significantly higher CD31-assessed MVD. Among 200 HNSCCs, Evans et al. [49] concluded that high CD31-assessed MVD was significantly associated with higher tumor stage and N-stage. Since it results in being specifically expressed on active endothelial cell surfaces, Endoglin (CD105) has been proposed as a reliable marker for MVD assessment in neoplasms [18]. Tanaka et al. [126] have showed that antibodies to CD105 react preferentially with active endothelial cells of angiogenic tissues in neoplasms; on the other hand, antibodies to pan-endothelial antigen (such as CD31 and CD34) may also react with stable vessels trapped in the tumor. In 71 OSCC, Chen et al. [44] found that patients with higher peripheral vein CD105 or venous return from tumor CD105 levels had poorer five-year disease-specific survival rate and OS.

#### 4.2.2. OSCC

Considering hypoxia signaling markers and using tissue microarrays of 66 patients with OSCC, dos Santos and colleagues [75] found that high HIF-1α expression was as-sociated with a reduction of DFS; independently of the adopted treatment, patients treated with post-operative radiotherapy had lower survival rates. In their attempt to individuate the optimal treatment approaches for stage I OSCC, Dunkel et al. [32] suggested that IHC for both CD44, the most important cell surface receptor for extra-cellular hyaluronan and a mediator of cell adhesion to its surroundings, and HIF-1α might be useful for the identification of patients with poor prognosis. A CD44-low, HIF-1α-high signature was associated with poorer DFS. A hypoxic microenvironment harbors both HIF-1α molecules and inflammatory cells. HIF-1α can interact with other inflammatory protein complexes, carrying out a crucial role in tumor inflammation’s regulation [148]. On the basis of inhibition of lymphoid cells in the peritumoral infiltrate, due to the interaction between cytokines and HIF-1α, results in a reduced tumor antigen recognition by lymphocytes [149]; this suggests that HIF-1α expression in tumor and peritumoral inflammatory cells could have an important prognostic role. In their investigation enrolling 56 patients with OSCC, they elaborated a risk profile defining the chance of disease relapse and death established on HIF-1α expression in tumoral inflammatory cells. This risk profile determined that high HIF-1α expression in peritumoral cells was associated with worse prognosis, independently of intra-tumoral expression. On the contrary, low HIF-1α levels in tumor margins and high expression in the tumor were associated with a low risk profile, with no cases of carcinoma recurrence or disease-related death. Among OSCC, the relationship between HIF-1α and well-established clinicopathological parameters has been hypothesized but different results were reported by various research groups. Choi et al. [74] did not find any correlation, whereas Bharti et al. [73] found an unexpected relationship between high HIF-1α expression and T1-staged tumors. However, no significant differences were disclosed for HIF-1α expression when compared with histological grade, clinical TNM higher stages, and nodal stage. Fillies et al. [131] found no association between HIF-1α expression and TNM stage in the floor of the mouth SCC. A possible explanation for such findings could be that HIF-1α expression is an early event in tumorigenesis, which is not hypoxia-related, but linked to alterations in tumor-suppressor genes and oncogenes [131]. Regarding nodal status in oral SCC, no positive association was found between HIF-1α and metastasis by Siriwardena et al. [86], but all highly expressing HIF-1α cases belonged to the pattern of invasion (POI) type IV. As POI type IV is associated with lymph node metastasis [150], the predominance of highly expressing HIF-1α cases in the POI type IV group may disclose an indirect association between HIF-1α expression and neck lymph node metastasis. Angiogenin (ANG) is an angiogenic factor that has been reported to induce tumor progression by stimulating both cancer cell proliferation and angiogenesis [151]. It is reasonably thought to be upregulated in a hypoxic environment through a HIF-1α-mediated mechanism. Kishimoto et al. [78] found an association between ANG and HIF-1α expression in OSCC specimens; both in vitro and in vivo tests showed that ANG was upregulated under hypoxic stimuli and was effectively related to cell proliferation and angiogenesis.

HIF-2α shares 48% of amino-acid sequence homology with HIF-1α, and its expression is restricted to specific cell types, including type II pneumocytes and endothelial cells. HIF-2α cooperates with its homologous but has different roles and seems to be associated with poor patient outcome in various tumors [152]. Lim et al. [80] hypothesized that HIF-2a overexpression could be a good biomarker for OSCC status for all tumor stages and could predict an early recurrence.

In OSCC, CA-IX plays different roles according to subcellular localization. Peterle et al. [85] identified an association between CA-IX membrane expression and DFS, whereas CA-IX’s strong cytoplasmic expression was associated with nodal metastases and disease-specific survival. In multivariate analysis, the positive CA-IX cytoplasmic expression retained its value as an independent risk factor for disease-related death. Besides CA-IX, the activation of the HIF-1 transcriptional complex promotes the expression of plasminogen activator inhibitor-1 (PAI-1), a member of the superfamily of serine-protease inhibitors (or serpins), which, according to the literature, has a paradoxical pro-tumorigenic role in cancer, promoting tumor cell survival and angiogenesis [153]. As for Ca-IX, PAI-1 immunopositivity was related with different findings in OSCC, according to subcellular localization [85]: membrane expression was an independent marker for local disease relapse, whereas a strong PAI-1 cytoplasmic immunopositivity was significantly associated with the less differentiated grading.

Adhesion molecules studied in HNSCC include CD44 and CD31. CD44 was associated with a good prognosis in early OSCC but, at the same time, a low expression of CD44 associated with a high expression of HIF-1α resulted in poor prognosis in advanced malignancies (75% of treated patients experienced recurrence or metastasis within 5 years) [32]. 

The CXC chemokine receptor 7 (CXCR7) binds to both CXCL12 (stromal derived growth factor, SDF1) and CXCL11 (IFN α chemo-acting inducible T cell, I TAC) and is involved in OSCC tumorigenesis [38,154]. In OSCC, the CXCR7 has been associated with aggressiveness and poor prognosis [38]. Its involvement in the angiogenetic process is confirmed by the fact that endothelial expression of CXCR7 is stimulated by VEGF and HIF-1α [155]. Moreover, it seems that in tumor endothelial cells angiogenesis is promoted by the autocrine axis CXCL12/CXCR7 through phosphorylation of the regulated extracellular signal kinase 1/2 (ERK1/2) [156]. Regarding the pro-inflammatory activity of CXCR7, it acts as a modulator of chemotactic activity of CXCL12; on the other hand, the expression of CXCR7 is enhanced by pro-inflammatory cytokines such as tumor necrosis factor (TNF) α and IL-1β [157]. All together, these features support the dual pro-inflammatory and premalignant role of CXCR7 in both the epithelial and endothelial cells of OSCC patients [38].

The common lymphatic and vascular endothelial receptor (CLEVER-1)/Stabilin-1 is expressed by lymphatic and inflamed vascular endothelium and type 2 macrophages [158]. Expression of CLEVER-1 promotes tumor growth and, being related to the polarization of the macrophage, is also considered a marker of tumor aggression in cervical lymph nodes [32].

IL-33, with its ST2 receptor, is involved in OSCC development. While IL-33 is ex-pressed by different cytotypes, including epithelial cells, endothelial ones, smooth muscle, fibroblasts, and activated macrophages, ST2 is expressed also by mast cells, Th2 cells, eosinophils, and basophils. IL-33 in the full-length, biologically active form is secreted when cells transmit inflammatory signals or undergo necrosis. IL-33, a powerful mast cell activator, also induces angiogenesis and vessel permeability. In tongue SCC, Ishikawa et al. [34] found that IL-33 expression correlated with mast cell density, supporting the hypothesis that IL-33 contributes to tumor progression by mast cells’ activation.

Analyzing 114 OSCC patients, Supic et al. [66] concluded that VEGF-A-1154GG genotype could be considered a prognostic marker of poor survival in advanced-stage tumors. Yanase et al. [70] evaluated VEGF-C in the surgical specimens from 61 patients with OSCC. They found that VEGF-C expression was associated with neck lymph node metastasis, carcinoma recurrence, and a poorer five-year survival rate. Multivariable analysis disclosed that VEGF-C was an independent prognostic factor for OSCC. Analyzing a series of 80 tongue carcinomas, Al-Shareef et al. [41] reported that there was an association between VEGF C expression levels and lymph node metastasis and DFS. Considering 90 patients with T1-2 N0 tongue SCC, Matsui et al. [60] concluded that VEGF-C expression was associated with neck node metastasis in a multiple logistic regression analysis. VEGFR-3 and angiopoietin-2 expression was determined by IHC in tumor tissues from 112 patients with OSCC by Li et al. [57]. High VEGFR-3 expression positively correlated with neck lymph node metastasis and lymphatic vessel density. In 2017, de Aquino et al. [45] investigated 50 lower lip SCCs and found that cytoplasmic expression of VEGFR-3 in the tumor core was associated with histological grade, metastasis, and patient death.

A positive association between non-nuclear maspin expression and angiogenesis regulation has been disclosed by Cho et al. [159] in the OSCC setting. Evaluating 33 OSCC cases, it was disclosed that cytoplasmatic maspin expression was negatively correlated with mutant-type p53 and VEGF, thus suggesting that the maspin gene could be a mutant-type p53 target in vivo and may contribute to downregulate VEGF expression. However, data from that series did not demonstrate a significant prognostic role of cytoplasmatic expression of maspin [159].

As previously reported, MMPs are important effectors downstream of the hypoxia biological cascade, whose prognostic role and complex biological interaction have been extensively described in OSCCs. In their retrospective clinical-pathological evaluation of 86 OSCCs, Lin et al. [100] showed that increased MMP-9 expression was associated with a more aggressive phenotype and the presence of neck lymph node metastasis. However, the available data seem to describe a complex picture in relation to different MMP types: MMP-7 cleaves type-XVIII collagen to endostatin and plasminogen to angiostatin, thus potentially inhibiting angiogenesis and, consequently, tumor growth [160]. In their retrospective evaluation on 61 OSCC cases, Mishev et al. [104] found that in high-stage tumors, MMP-7 expression levels decreased, reflecting the fact that, as the malignancy potential increased, the ability of MMP-7 to retard its invasion considerably declined. On the other hand, the same investigation found that MMP-2 and MMP-9 levels remained constant across OSCC stages, arguing that they could not be used as suitable surrogate marker of tumor-invasiveness potential [104].

Prostate-specific membrane antigen (PSMA) is a type of MMP which was firstly found expressed on tumor endothelial cells in prostate carcinoma; its peptidase function is supposed to contribute to endothelial cell invasion [161]. In their retrospective im-immunohistochemical evaluation of 96 OSCCs, Haffner and colleagues [93] found that higher PSMA levels were highly associated with a worse OS. ADAM 10, a disintegrin and metalloproteinase protein, appeared to be highly expressed in OSCC, especially in metastatic disease; it is also associated with increased MVD and, therefore, reflects angiogenesis activation [106].

Regarding the complex biological interaction between cancer cells and tumor microenvironment, cancer-associated myofibroblasts represent crucial mesenchymal cells, playing numerous roles in promoting tumor growth, invasion, and metastasis [162]. Myofibroblast proliferation in the microenvironment is involved in the epithelial–mesenchymal transition (EMT) in HNSCC, thus causing tumor invasion, the occurrence of occult neck lymph nodes disease, distant metastasis, and poor survival in OSCC [100]. Smooth Muscle Actin (SMA), which belongs to the highly conserved actin family, is expressed in myofibroblasts, which play a role in cell motility, structure, and integrity. Alpha-, β- and γ-actin isoforms have been identified. In particular, SMA is an α-actin that is found in skeletal muscle, on smooth muscle vessel walls, myometrium, the gut wall, and myoepithelial cells in breast and salivary glands. α-SMA appear in stress fibers of fibroblastic cells during pathological situations [163]. Analyzing the prognostic role of cancer-associated myofibroblasts, considerable evidence has shown that the expression of their marker, α-SMA, was significantly higher in OSCC, particularly in moderately differentiated carcinomas and in metastatic lesions [50]. Another study by Lin et al. [100] reported a significant association between elevated α-SMA levels and increased risk of tumor and lymph node invasion and recurrence in OSCC. Maqsood et al. [101] reported that α-SMA was present in stromal cells of the host tissue as a response to tumoral invasion and is also highly expressed specifically in poorly differentiated OSCC.

Kämmerer et al. [53] immunostained 50 OSCC samples with CD31-antibodies. A significantly higher MVD was found in T3-T4 vs. T1-T2, N+ vs. N0, as well as G3–G4 vs. G1–G2 OSCCs. A higher CD31-assessed MVD was also associated with increased and earlier rates of local recurrence, more metastases, and significantly decreased OS and DFS.

#### 4.2.3. LSCC

A Chinese research group [79] evaluated HIF-1α and survivin expression in LSCC tissues from all stages and cell lines. HIF-1α and survivin were both highly expressed in neoplastic tissue and related to the clinical stage and neck nodes metastases, with a positive correlation between the expression of the two markers. In in vitro analyses, hypoxic stress significantly increased HIF-1α and survivin expression levels; in hypoxic cells, the downregulation of HIF-1α expression decreased survivin gene expression. According to the data of Li et al. [79], HIF-1α could be considered a regulating factor for survivin gene expression in laryngeal SCC cells under hypoxic conditions; both proteins could be considered predictors of malignancy progression in LSCC. Three series including patients with laryngeal and hypopharyngeal SCC at different stages failed to attribute a potential prognostic [84] or predictive role of response to chemo/radiotherapy [72,81] to HIF-1α.

Regarding CA-IX, another hypoxia-related factor, its significance in LSCC varies in relation to stage. In 2013, Wachters et al. [164] did not find any prognostic significance for CA-IX towards local control in supraglottic T1-T2 SCC. More recently, the same research group [88] concluded that lower CA-IX expression was evident in supraglottic low-stage laryngeal SCCs, without any prognostic relevance. Lack of prognostic effect of CA-IX was found in a series of 286 early glottic SCCs treated with radiotherapy [76]. Differences in clinicopathological and immunohistochemical staining results for HIF-1α and CA-IX support the hypothesis that early stage glottic and supraglottic LSCCs could be different entities [88]. In their study focusing specifically on T3 and T4 laryngeal and hypopharyngeal SCCs, Bernstein et al. [72] found that CA-IX expression might confer a more aggressive tumor phenotype, as pre-treatment immunohistochemical CA-IX expression was identified as an adverse prognostic factor for disease-specific survival.

The CXCR2 is a member of the seven-transmembrane domain rhodopsin-like G protein-coupled receptors, known to have a role in immune response processes, chronic inflammation, and sepsis, as well as in tumorigenesis, angiogenesis, and metastases of several human cancers [33]. Overexpression of CXCR2 in laryngeal SCC patients leads to a worse prognosis [33]. CXCR2 angiogenetic activity lies in the binding of the receptor with chemokines presenting the ERL (glutamic acid-leucine-arginine)-CXC chemokines, among which IL-8; ENA-78; GRO-a, -b, and -c; neutrophil-activating protein-2 (NAP-2)-a have been reported in several tumors’ development [33]. 

To confirm the role of fibroblast growth in modulating the tumor microenvironment, Starska et al. [106] found that the fibroblast growth factor receptors, FGFR1 and FGFR3, and the pathway downstream of its regulatory kinases phosphoinositide 3-kinase (PI3K)/AKT promoted the invasiveness of cancer cells resulting in a worse prognosis of laryngeal cancer.

Erkılınç et al. [90] identified an association between PSMA expression on tumoral vascular endothelium and prognosis in LSCC; elevated marker levels were recorded in the case of cartilage or local invasion and in a more advanced stage of the disease.

In LSCC, the interaction between the angiogenetic pathway and immune microenvironment has been only very recently investigated. In LSCC, two proteins that may be overexpressed on tumor cells are the transmembrane glycoprotein programmed death 1 (PD-1) and its death-ligand programmed ligand 1 (PD-L1) [165,166]. PD-L1 is a type I transmembrane glycoprotein in the B7 superfamily. It is generally expressed on several cell type’s membranes, but it may also be overexpressed in tumor cells. When PD-L1 binds to PD-1 (a transmembrane protein expressed on the T-cell membrane), an inhibitory signaling cascade develops in the T lymphocyte, blocking its immune response [165,167]. Our research group assessed 45 consecutive cases of laryngeal SCC CD31-assessed MVD, PD-L1 in terms of combined positive score (CPS) and tumor infiltrating lymphocytes (TILs) [166]. Cox proportional hazards model found increasing CD31-assessed MVD values, PD-L1 CPS < 1, and TILs count rate < 30%, as predictive of reduced DFS. Our multivariable analysis disclosed that MVD and TILs retained their independent prognostic value. In HNSCC, the angiogenetic activity of VEGF seems to interact directly with the innate and adaptive pathways of immune response, inhibiting the NF-kB cascade, which in turn regulates the expression of PD-L1, thus affecting the function of the immune checkpoint [165,166].

Concerning the cell adhesion molecules, the overexpression of CD31 together with VEGF was related to worse prognosis in early-stage LSCC [64,166].

CD105 is a disulfide-linked, proliferation-associated, hypoxia-inducible cell membrane glycoprotein. It is a component of the receptor complex of transforming growth factor-β (TGF-β), a cytokine which modulates angiogenesis by regulating various cell functions, including differentiation, proliferation, and migration [168]. CD105 is involved in activating a complex signaling pathway conditioning the proliferation, migration, and adhesion of endothelial cells, resulting in increased angiogenesis. Very recently, investigating the prognostic role of CD105- and CD31-assessed MVD in paired LSCC biopsies and surgical specimens, our research group concluded that using CD31 led to an MVD overestimation with respect to CD105. In fact, CD31 was often expressed in tumor micro-vessels in samples showing no CD105 staining [169]. Since it appears to be specifically expressed on the surface of active endothelial cells, CD105 has been reported as a reliable marker for HNSCC MVD assessment [18]. Marioni et al. [151] evaluated the relationship between CD105 expression on 108 consecutive, operable LSCCs. Higher loco-regional carcinoma recurrence rate and lower DFS were found in cases with high CD105 expression. 

The ability of CD105 to quantify MVD in HNSCC has allowed the role of different oncogenes, tumor suppressors, and pathways in neoangiogenesis to be explored. Survivin—a member of the family of inhibitor of apoptosis proteins that control cell division, apoptosis, and metastasis—is overexpressed in virtually all human cancers, including LSCC. Our clinical research group ascertained nuclear survivin expression and CD105-assessed MVD in 75 LSCCs by image analysis [170]. There was a strong positive correlation between nuclear survivin expression and MVD. The odds ratio (OR) for recurrence was approximately 2.8 in patients with a nuclear survivin expression ≥6.0% and 12.3 in those with an MVD ≥6.89%. One of the pathways controlled by EGFR involves the mammalian target of rapamycin (mTOR), a proto-oncogene activated in several cellular functions [171]. Our group studied the expression of mTOR and EGFR in LSCC cells and their correlation with tumor neo-angiogenesis, in terms of CD105-assessed MVD, and prognosis [172]. There was a strong positive correlation between mTOR and EGFR expression and between mTOR and MVD. Patients with a CD105-assessed MVD >5.28% had a higher recurrence rate and a lower DFS. In multivariable analysis, only N stage and CD105-assessed MVD maintained their independent prognostic significance in terms of DFS. The same factors that drive epithelial cells toward a mesenchymal phenotype may also drive endothelial cells toward a pro-angiogenic phenotype. In 2021, Franz et al. [17] investigated a potential interplay between EMT and angiogenesis in LSCC (quantified through CD105 expression). A trend toward a significant correlation was reported between two EMT markers (Snail and Zeb2) and CD105-assessed MVD. Very recently, Alessandrini et al. [173] analyzed tumor–stroma ratio (TSR) and CD31- and CD105-assessed MVD in paired biopsies and surgical specimens of 43 consecutive LSCC cases. Considering biopsies, multivariate analysis found both TSR- and CD105-assessed MVD as DFS predictors. In paired surgical specimens, both TSR- and CD105-assessed MVD retained their significance in multivariate analyses. Considering a cohort of 89 surgically-treated LSCCs, CD105 > 6% and Nm23-H1 expression < 10% were significantly associated with malignancy recurrence, retaining their independent prognostic values even in the multivariate regression model [91]. In a study on LSCC, Zhou et al. [112] also found that levels of nm23-H1 expression in recurrent patients were significantly lower than those in non-recurrent patients, and the low expression level of nm23-H1 was an independent risk factor for recurrence. Regarding the prognostic value of onco-suppressor maspin in the specific setting of LSCCs, its expression at the level of cancer cell nucleus has been reported to be predictive of a more favorable outcome [174], probably counterbalancing pro-proliferative and pro-angiogenetic stimuli. Moreover, in LSCC, maspin nuclear localization was found to be related to reduced density of tumor-associated CD105-assessed micro-vessels [174]. On the other hand, in LSCC, non-nuclear maspin expression was not significantly associated with tumor recurrence per se; it might be considered in biomarkers panels, identifying combined expression patterns with a potential prognostic role. In this sense, Franz et al. [91] found that a cluster of LSCC patients with high expression of CD105 and non-nuclear maspin as well as low expression of nm23-H1 were significantly correlated with an increased risk of recurrence. Maspin participation in other biological signaling pathways was investigated. In a series 79 consecutive cases of surgically treated LSCCs, our research group found that maspin subcellular localization (nuclear vs. cytoplasmatic) affected the prognostic role of mTOR [102]. In fact, mTOR expression was associated with higher recurrence rate in cases with cytoplasmatic maspin, whereas such an association was not evident in those with nuclear maspin; thus, a tumor-suppressant role of nuclear maspin that compensated for the pro-proliferative effect of mTOR can be hypothesized [102].

#### 4.2.4. Pharyngeal SCC

The prevalence of proven HPV infection in oropharyngeal cancers has been increasing for decades in the Western world, accounting for more than 70% of cases [175]. Despite being associated with a more favorable prognosis, survival rates for patients with HPV-positive oropharyngeal cancers remain at approximately 80% at 3 years [176]. Biological and morphological differences are apparent between HPV-associated and HPV-unrelated oropharyngeal SCC: the former occurs at a younger age, has a better response to (chemo-) radiotherapy and better overall survival (OS), and is more likely to have a higher stage than the latter [77]; higher stages are associated with hypoxia. According to the following few studies, prognostic significance of HIF-1α seems strong in oropharyngeal SCCs, irrespective of HPV status. High HIF-1α expression was an independent risk factor for poor prognosis for advanced human-papillomavirus-unrelated oro- and hypo-pharyngeal cancer in the series by Agena et al. [71]. In their series of 233 oropharyngeal SCCs, Hong et al. [176] detected a HIF-1α immuno-positivity rate of approximately 59%; HIF-1α positivity was significantly associated with higher T category (T3/T4 vs. T1/T2, 64.2% vs. 48.4%) and lower pathological grading (Grade 1–2 vs. 3, 62% vs. 46.9%). No other significant association between HIF-1α ex-pression and HPV status or between HIF-1α and clinical outcome emerged from their data.

Moreover, the HPV-driven carcinogenesis is a complex process in which several pro-inflammatory stimuli interact within the overall framework of the local microbiome. In fact, an oral and oropharyngeal dysbiosis may lead to the summation and the interaction of the HPV-related transformation processes with the broad spectrum of inflammatory damages caused by several pathogenic agents, including C. albicans, which is the most relevant cancer-associated fungus in the head and neck [177]. This has been advocated as a crucial pathogenetic issue especially in pediatric cases [178], which are less exposed to the traditional carcinogenic agents related to oral and oropharyngeal SCCs in the adult population.

Inflammatory microenvironment can determine an accumulation of cell mutations leading to tumor development. Several molecules including cytokines, cellular adhesion proteins, growth factors, and other peptides and immunomodulators are involved in this process. Ang-2 is the active peptide of the renin-angiotensin system (RAS), whose receptors are AT1 and AT2. The Ang-2/AT1 binding leads to various effects, including inflammation, fibrosis, and angiogenesis. Because the effects of AT1 and AT2 binding are often antagonistic, the AT2 receptor is usually activated under pathological conditions [179,180]. In esophageal cancer, Ang-2 is primarily expressed in the areas of inflammation, tissue damage, and metastasis, in addition to the neovascular areas. In general, an Ang-2 overexpression occurs during initial events in cancer development to remain constant in the middle and late stages. It is involved in the infiltration process as it induces activation of associated cytokines, including MMP. It promotes invasion and tumor progression acting together with VEGF expression and ultimately has a role in the angiogenetic process when it is expressed by endothelial cells inducing the development of new blood vessels to rebuild tumor micro-vascularization [26]. In hypopharyngeal carcinoma, the expression of MMP-9 is positively related to the angiogenetic process. The MMP-9 capacity to stimulate tumor invasion and metastatic formations of the cervical lymph nodes was demonstrated in tongue SCC [26].

Interleukins are considered biomarkers of poor prognosis and even of possible early development of relapses in oropharyngeal and esophageal SCCs [181]. Interleukins can induce MMP 9 overexpression, which promotes both angiogenesis and the process of tumor invasion and metastases by stimulating infiltration and recruitment of Th cells and other inflammatory cells [182]. The tumor microenvironment can be affected by various interleukins such as factor IL-6, which has a role in tumorigenesis and development [183], and factor IL-1, which is involved in the angiogenetic and lymph-angiogenetic process, acting together with VEGF and FGF [26,184]. The IL-1β is able to stimulate the secretion of IL-6 by fibroblasts, which in turn promotes tumor angiogenesis, metastasis, and invasion through EMT [185,186], demonstrating a direct correlation with the neovascularization process [26].

Transforming Growth Factor-β (TGF-β) exerts an inhibitory effect in the initial stage of cancer development; in particular, it acts on the cell cycle and activates the apoptotic process [187]. On the contrary, in advanced stages, it has a role in inducing invasion process and metastatic activity by stimulating the proliferation of the ECM and promoting angiogenesis and immunosuppression [188].

CXCL11 is a chemokine with a chemo-attractive function that plays a key role in the migration of cells. In the tumor microenvironment, I-TAC promotes tumor invasion and metastasis [154]. In HNSCC, its angiogenetic action is manifested by acting together with VEGF, where the cancer cells themselves produce and release I-TAC by acting on the surrounding inflammatory cells [189]. The I-TAC is also capable of binding the CXCR3, leading to both pro- and anti-tumor activities [26,190].

CD31 is a platelet endothelial cell adhesion molecule, which is why it is also known as PECAM-1. CD31 leads to the activation of immunity cells, such as leukocytes, monocytes, and neutrophils; meanwhile, it exerts an anti-angiogenetic effect through its cell–cell adhesion activity, enabling new vascularization. In primary cancer tissues of patients with unresectable oropharyngeal SCC, its down-expression appears directly related to a favorable prognosis condition when associated together with low levels of p21, p27, and Ki-67 and high levels of p53, cyclin D1, and EGFR [36].

Hong et al. [51] considered 50 cases of hypopharyngeal SCC and concluded that CD105-assessed MVD differed significantly across different pathological grades and T stages and regarding the presence of neck lymph node metastasis.

### 4.3. Early Detection and Screening—Predictive Markers

#### 4.3.1. LSCC

In early stage LSCC, radiotherapy is a major treatment modality. Radiotherapy failure can result in highly morbid salvage surgery, which could be prevented only by finding new predictors of radiotherapy efficacy besides TNM staging. However, HIF1-α does not seem to be an eligible candidate marker in this field, according to the studies by Wachters et al. [88,164] and by Douglas et al. [76].

Both FGFRs and their ligands, fibroblast growth factors (FGFs), are involved in various normal biological processes including cellular differentiation, proliferation, growth, survival, migration, and angiogenesis [191,192]. Starska et al. [106] analyzed FGFR1 and FGFR3 mRNA/protein levels in LSCC, observing that they were significantly associated; an increase in FGFR1 and FGFR3 expression was found in more than 70% of LSCCs, and these markers were related to tumor invasion, loco-regional control, and clinical outcomes.

#### 4.3.2. Pharyngeal SCC

Different results in terms of HIF-1α significance were found by Swartz et al. [87,193], who established an association between HIF-1α overexpression and a worse OS. However, the effect of HIF-1α overexpression on OS was lower in HPV-negative than in HPV-positive malignancies. The clinical relevance of these data relies on treatment deintensification for patients with HPV-positive tumors [194,195]. Therefore, treatment deintensification could not be appropriate for the sub-group of HPV-positive oropharyngeal SCCs with HIF-1α overexpression, due to their unfavorable prognosis similar to HPV-unrelated cancers. In HPV-associated oropharyngeal SCC, Swartz et al. [193] observed that CA-IX overexpression was associated with worse OS in chemoradiotherapy/radiotherapy-treated patients, regardless of HIF-1α, despite HIF-1α regulating transcription of CA-IX. CA-IX expression is regulated by HIF-1α through hypoxia-responsive elements located directly upstream of the promotor region of the gene coding for CA-IX, but it is also regulated through other regulatory pathways [196].

### 4.4. Treatment and Monitoring—Markers with Potential Therapeutic Role

#### HNSCC

As described above, angiogenesis mechanisms in HNSCC offer a wide range of biological processes to be potentially targeted by therapy. Considering a well-established biological marker such as EGFR, therapeutic agents targeted against it have been employed in treatment of advanced HNSCCs. The anti-EGFR agent Cetuximab has long been used to treat recurrent or metastatic HNSCCs either as a single agent in primary concurrent chemo-radiation regimens [197] or in combination with docetaxel in the postoperative adjuvant setting [198]. However, such indications for Cetuximab have been considered by the NCCN Guidelines for the treatment of HNSCC to be based on a relatively low evidence level (category 2B) [199]. On the other hand, stronger evidence supports the use of Cetuximab in association with platinum-based regimens for recurrent, unresectable, or metastatic HNSCC [200,201,202], leading to a strong recommendation (category 1) of such therapeutic approaches by the NCCN Guidelines in these settings [199].

Regarding therapies directly targeted against angiogenetic effectors, no agent is currently part of the clinical practice for these patients because conclusive data from clinical trials on such drugs in HNSCC setting are still unavailable [203,204,205]. Limited evidence on the efficacy of the use of the anti-VEGFR agent bevacizumab is available to date for HNSCC therapy [206,207]. A recent phase III trial comparing bevacizumab (in association with platinum-based regimens) with conventional chemotherapy [207] found an improvement in progression-free survival in the bevacizumab arm; however, it is associated with a higher toxicity rate. A combination of bevacizumab with radiation therapy has long been tested in experimental conditions with various tumor models, showing a promising synergistic effect [208]. However, evidence regarding bevacizumab in combination with radiotherapy and traditional chemotherapies are sparse and heterogeneous in the HNSCC setting [209,210,211].

Targeting multiple angiogenesis pathways with multi-kinase inhibitors has long gained investigators’ attention in various cancer settings. However, evidence regarding the use of such drugs, including sunitinib and semaxinib, to treat HNSCCs is limited and seems to not show clear anti-tumor effectiveness [203,204,212]. As a result, such therapeutic agents are not currently considered among the standards of care for HNSCCs [198]. Lenvatinib, a multikinase inhibitor against VEGFR1-3, platelet-derived growth factor receptor-a (PDGFR-a), c-Kit, and the RET, already approved to treat several solid cancers including thyroid cancer, renal cell and hepatocellular carcinomas, has showed promising outcomes and a manageable safety profile in HNSCC treatment [213]. However, this approach is currently still investigational and needs to be supported by further evidence [203].

Endoglin itself has been regarded as a potential target alternative to VEGFR [214]. The most studied anti-endoglin drug is TRC105, a monoclonal antibody targeted against the extracellular domain of endoglin. Investigations with this antibody have shown that it not only affects angiogenesis, but also reduces circulating Treg cells [215] and even targets cancer-associated fibroblast as well as other CD105+ cells within the tumor microenvironment [216]. Such therapy seems to potentially reduce circulating tumor cells and the generation of metastases in different cancer types [217,218]. However, clinical evidence specific for the HNSCC setting still needs to be obtained.

A suggestive therapeutic development in HNSCC may be the combination of anti-angiogenic and immunotherapeutic drugs. In other cancer types including advanced hepatocellular carcinoma, such an approach has shown promising results, showing a significant survival improvement [219,220,221]. The rational underlying such an approach may reside in the idea of vascular normalization, achieved by low-dose antiangiogenic drugs. These drugs may potentially normalize tumor blood vessels, thereby facilitating the infiltration of effector T cells and possibly reducing the toxicity profile, which is associated with standard-dose anti-angiogenetic drugs [222].

## 5. Conclusions

Several signals capable of tripping the “angiogenic switch” have also been identified in HNSCC. The following concepts should be considered as “take home messages” in the context of the role that current knowledge on angiogenesis in HNSCC could play in future clinical use. Anti-angiogenic agents with differing mechanisms of action have been tested in the treatment of HNSCC with mixed success [204]. Although several studies suggest that anti-angiogenic agents might be a valuable adjunct to conventional chemoradiation of HNSCC, their long-term therapeutic value remains uncertain [223]. Further investigations on combinations of anti-angiogenic agents with conventional chemotherapeutic ones, immunotherapeutic, and molecularly targeted agents in HNSCC are necessary. In particular, in view of a tailored therapy, further data are needed to pinpoint the patients who will benefit most from these treatments.

## Data Availability

The data presented in this study are available on request from the corresponding author.

## Supplementary Materials

The supporting information can be downloaded at: https://www.mdpi.com/article/10.3390/ijms241310733/s1.

## Appendix A. Search Queries

((head and neck) OR (oral cavity) OR (oropharynx) OR (nasopharynx) OR (larynx) OR (hypopharynx) OR (salivary gland)) AND (squamous cell) AND ((cancer) OR (carcinoma) OR (tumor) OR (malignancies)) AND ((angiogenesis) OR (neoangiogenesis) OR (cd105) OR (cd31) OR (endoglin) OR (vegf) OR (vegfr) OR (CD34) OR (angiogenin) OR (hypoxia-inducible factor 1) OR (HIF-1) OR (Galectin microvessel density) OR (MVD)).

## References

[1] Mete O., Wenig B.M. (2022). Update from the 5th Edition of the World Health Organization Classification of Head and Neck Tumors: Overview of the 2022 WHO Classification of Head and Neck Neuroendocrine Neoplasms. Head Neck Pathol..

[2] Gatta G., Capocaccia R., Botta L. (2023). Descriptive epidemiology of the head and neck cancers in old patients. Front. Oncol..

[3] Gatta G., Botta L., Sánchez M.J., Anderson L.A., Pierannunzio D., Licitra L. (2015). EUROCARE working group: Prognoses and improvement for head and neck cancers diagnosed in Europe in early 2000s: The EUROCARE-5 population-based study. Eur. J. Cancer.

[4] Capote A., Escorial V., Muñoz-Guerra M.F., Rodríguez-Campo F.J., Gamallo C., Naval L. (2007). Elective neck dissection in early-stage oral squamous cell carcinoma—Does it influence recurrence and survival?. Head Neck.

[5] Kowalski L.P., Bagietto R., Lara J.R., Santos R.L., Silva J.F., Magrin J. (2000). Prognostic significance of the distribution of neck node metastasis from oral carcinoma. Head Neck.

[6] Pulte D., Brenner H. (2010). Changes in survival in head and neck cancers in the late 20th and early 21st century: A period analysis. Oncologist.

[7] Lothaire P., de Azambuja E., Dequanter D., Lalami Y., Sotiriou C., Andry G., Castro G., Awada A. (2006). Molecular markers of head and neck squamous cell carcinoma: Promising signs in need of prospective evaluation. Head Neck.

[8] Lopes M.A., Nikitakis N.G., Reynolds M.A., Ord R.A., Sauk J. (2002). Biomarkers predictive of lymph node metastases in oral squamous cell carcinoma. J. Oral Maxillofac. Surg..

[9] Chen C., Wang Z., Ding Y., Qin Y. (2023). Tumor microenvironment-mediated immune evasion in hepatocellular carcinoma. Front. Immunol..

[10] Johnson D.E., Burtness B., Leemans C.R., Lui V.W.Y., Bauman J.E., Grandis J.R. (2020). Head and neck squamous cell carcinoma. Nat. Rev. Dis. Prim..

[11] Folkman J. (2002). Role of angiogenesis in tumor growth and metastasis. Semin. Oncol..

[12] Franz L., Alessandrini L., Fasanaro E., Gaudioso P., Carli A., Nicolai P., Marioni G. (2021). Prognostic impact of neutrophils-to-lymphocytes ratio (NLR), PD-L1 expression, and tumor immune microenvironment in laryngeal cancer. Ann. Diagn. Pathol..

[13] Alessandrini L., Franz L., Ottaviano G., Ghi M.G., Lanza C., Blandamura S., Marioni G. (2020). Prognostic role of programmed death ligand 1 (PD-L1) and the immune microenvironment in laryngeal carcinoma. Oral Oncol..

[14] Alessandrini L., Franz L., Sbaraglia M., Saccardo T., Cappello F., Drigo A., Frigo A.C., Marioni G. (2022). Tumor-Stroma Ratio and Programmed Cell Death Ligand 1 Expression in Preoperative Biopsy and Matched Laryngeal Carcinoma Surgical Specimen. Int. J. Mol. Sci..

[15] Yao C., Wu S., Kong J., Sun Y., Bai Y., Zhu R., Li Z., Sun W., Zheng L. (2023). Angiogenesis in hepatocellular carcinoma: Mechanisms and anti-angiogenic therapies. Cancer Biol. Med..

[16] Dzobo K., Senthebane D.A., Dandara C. (2023). The Tumor Microenvironment in Tumorigenesis and Therapy Resistance Revisited. Cancers.

[17] Franz L., Nicolè L., Frigo A.C., Ottaviano G., Gaudioso P., Saccardo T., Visconti F., Cappellesso R., Blandamura S., Fassina A. (2021). Epithelial-to-mesenchymal transition and neoangiogenesis in laryngeal squamous cell carcinoma. Cancers.

[18] Franz L., Alessandrini L., Saccardo T., Frigo A.C., Marioni G. (2020). CD105- and CD31-assessed microvessel density in laryngeal carcinoma biopsies as a predictor of recurrence after exclusive primary surgery. Ann. Diagn. Pathol..

[19] Mermod M., Bongiovanni M., Petrova T., Goun E., Simon C., Tolstonog G., Monnier Y. (2019). Prediction of occult lymph node metastasis in head and neck cancer with CD31 vessel quantification. Otolaryngol. Neck Surg..

[20] Dong Y., Ma G., Liu Y., Lu S., Liu L. (2020). Prognostic Value of Microvessel Density in Head and Neck Squamous Cell Carcinoma: A Meta-Analysis. Dis. Markers.

[21] Guo X., Meng X., Liu R. (2021). Prognostic value of microvessel density in esophageal squamous cell carcinoma-a systematic review and meta-analysis. Pathol. Res. Pract..

[22] Kampoli K., Foukas P.G., Ntavatzikos A., Arkadopoulos N., Koumarianou A. (2022). Interrogating the interplay of angiogenesis and immunity in metastatic colorectal cancer. World J. Methodol..

[23] Cai C., Wang X., Fu Q., Chen A. (2022). The VEGF expression associated with prognosis in patients with intrahepatic cholangiocarcinoma: A systematic review and meta-analysis. World J. Surg. Oncol..

[24] Bhardwaj V., Zhang X., Pandey V., Garg M. (2023). Neo-vascularization-based therapeutic perspectives in advanced ovarian cancer. Biochim. Biophys. Acta Rev. Cancer.

[25] Vassilakopoulou M., Psyrri A., Argiris A. (2015). Targeting angiogenesis in head and neck cancer. Oral Oncol..

[26] Guo W., Yin G., Liu H., Duan H., Huang Z. (2020). Analysis of vascular-associated factors and the prognosis of poorly differentiated hypopharyngeal carcinoma. Oncol. Lett..

[27] Viallard C., Larrivée B. (2017). Tumor angiogenesis and vascular normalization: Alternative therapeutic targets. Angiogenesis.

[28] Zhan J., Zhang M., Zhou L., He C. (2023). Combination of immune checkpoint blockade and targeted gene regulation of angiogenesis for facilitating antitumor immunotherapy. Front. Bioeng. Biotechnol..

[29] Page M.J., McKenzie J.E., Bossuyt P.M., Boutron I., Hoffmann T.C., Mulrow C.D., Shamseer L., Tetzlaff J.M., Akl E.A., Brennan S.E. (2021). The PRISMA 2020 statement: An updated guideline for reporting systematic reviews. PLoS Med..

[30] NHLBI Study Quality Assessment Tools. www.nhlbi.nih.gov/health-topics/study-quality-assessmenttools.

[31] Aggarwal S., Sharma S.C., Das S.N. (2015). Galectin-1 and galectin-3: Plausible tumour markers for oral squamous cell carcinoma and suitable targets for screening high-risk population. Clin. Chim. Acta.

[32] Dunkel J., Vaittinen S., Koivunen P., Laranne J., Mäkinen M.J., Tommola S., Irjala H. (2016). Tumoral Expression of CD 44 and HIF 1α Predict Stage I Oral Cavity Squamous Cell Carcinoma Outcome. Laryngoscope Investig. Otolaryngol..

[33] Han L., Jiang B., Wu H., Wang X., Tang X., Huang J., Zhu J. (2012). High expression of CXCR2 is associated with tumorigenesis, progression, and prognosis of laryngeal squamous cell carcinoma. Med. Oncol..

[34] Ishikawa K., Yagi-Nakanishi S., Nakanishi Y., Kondo S., Tsuji A., Endo K., Wakisaka N., Murono S., Yoshizaki T. (2014). Expression of interleukin-33 is correlated with poor prognosis of patients with squamous cell carcinoma of the tongue. Auris Nasus Larynx.

[35] Kayamori K., Katsube K., Sakamoto K., Ohyama Y., Hirai H., Yukimori A., Ohata Y., Akashi T., Saitoh M., Harada K. (2016). NOTCH3 Is Induced in Cancer-Associated Fibroblasts and Promotes Angiogenesis in Oral Squamous Cell Carcinoma. PLoS ONE.

[36] Soba E., Budihna M., Smid L., Gale N., Lesnicar H., Zakotnik B., Strojan P. (2015). Prognostic value of some tumor markers in unresectable stage IV oropharyngeal carcinoma patients treated with concomitant radiochemotherapy. Radiol. Oncol..

[37] Tokmak S., Arık D., Pınarbaşh Ö., Gürbüz M.K., Açıkahn M.F. (2021). Evaluation and Prognostic Significance of Galectin-3 Expression in Oral Squamous Cell Carcinoma. Ear Nose Throat J..

[38] Yanagiya M., Dawood R.I.H., Maishi N., Hida Y., Torii C., Annan D.A., Kikuchi H., Yanagawa Matsuda A., Kitamura T., Ohiro Y. (2021). Correlation between endothelial CXCR7 expression and clinicopathological factors in oral squamous cell carcinoma. Pathol. Int..

[39] Yang Q., Liu Y., Huang Y., Li Y., Wu J., Duan M. (2013). Expression of COX-2, CD44v6 and CD147 and Relationship with Invasion and Lymph Node Metastasis in Hypopharyngeal Squamous Cell Carcinoma. PLoS ONE.

[40] Aggarwal S., Devaraja K., Sharma S.C., Das S.N. (2014). Expression of vascular endothelial growth factor (VEGF) in patients with oral squamous cell carcinoma and its clinical significance. Clin. Chim. Acta.

[41] Al-Shareef H., Hiraoka S.I., Tanaka N., Shogen Y., Lee A.D., Bakhshishayan S., Kogo M. (2016). Use of NRP1, a novel biomarker, along with VEGF-C, VEGFR-3, CCR7 and SEMA3E, to predict lymph node metastasis in squamous cell carcinoma of the tongue. Oncol. Rep..

[42] Ansari F., Asif M., Kiani M., Ara N., Ishaque M., Khan R. (2020). Evaluation of Mast Cell Density using CD117 antibody and Microvessel Density Using CD34 Antibody in Different Grades of Oral Squamous Cell Carcinoma. Asian Pac. J. Cancer Prev..

[43] Bertini F., do Prado R.F., de Oliveira A.J., Tera T.M., Rosa L.E.B., Seabra J.C., Carvalho Y.R. (2016). Correlation between blood and lymphatic microvascular density and cell proliferation in mouth floor and tongue squamous cell carcinoma. J. Oral Maxillofac. Surg. Med. Pathol..

[44] Chen C.H., Chuang H.C., Lin Y.T., Fang F.M., Huang C.C., Chen C.M., Lu H., Chien C.Y. (2016). Circulating CD105 shows significant impact in patients of oral cancer and promotes malignancy of cancer cells via CCL20. Tumor Biol..

[45] De Aquino A.R.L., Nonaka C.F.W., de Carvalho C.H.P., Demeda C.F., de Souza L.B., Pinto L.P. (2017). Immunoexpression of VEGFR-3, but not the immunoexpression of VEGF-C or lymphatic density, is correlated with metastasis in lower lip squamous cell carcinoma. Int. J. Oral Maxillofac. Surg..

[46] De Oliveira M.V.M., Pereira Gomes É.P., Pereira C.S., de Souza L.R., Barros L.O., Mendes D.C., Guimarães A.L., De Paula A.M. (2013). Prognostic value of microvessel density and p53 expression on the locoregional metastasis and survival of the patients with head and neck squamous cell carcinoma. Appl. Immunohistochem. Mol. Morphol..

[47] De Sousa E.A., Lourenço S.V., de Moraes F.P., Vartanian J.G., Gonçalves-Filho J., Kowalski L.P., Soares F.A., Coutinho-Camillo C.M. (2015). Head and neck squamous cell carcinoma lymphatic spread and survival: Relevance of vascular endothelial growth factor family for tumor evaluation: Lymphangiogenesis in HNSCC. Head Neck.

[48] Etemad-Moghadam S., Alaeddini M. (2019). Upregulation of ADAM10 in oral squamous cell carcinoma and its correlation with EGFR, neoangiogenesis and clinicopathologic factors. J. Craniomaxillofac. Surg..

[49] Evans M., Baddour H.M., Magliocca K.R., Müller S., Nannapaneni S., Chen A.Y., Kim S., Chen Z., Shin D.M., Wang A.Y. (2019). Prognostic implications of peritumoral vasculature in head and neck cancer. Cancer Med..

[50] Gadbail A.R., Korde S., Chaudhary M.S., Sarode S.C., Gondivkar S.M., Dande R., Tekade S.A., Yuwanati M., Hande A., Patil S. (2020). Ki67, CD105, and α-SMA expression supports biological distinctness of oral squamous cell carcinoma arising in the background of oral submucous fibrosis. Asian Pac. J. Cancer Prev..

[51] Hong Y.M., Gan W.G., Xu Z.H. (2014). Significance of the expression of integrin β1, VEGF and MVD in hypopharyngeal squamous cell carcinoma. Genet. Mol. Res..

[52] Jung S., Sielker S., Purcz N., Sproll C., Acil Y., Kleinheinz J. (2015). Analysis of angiogenic markers in oral squamous cell carcinoma-gene and protein expression. Head Face Med..

[53] Kämmerer P.W., Al-Nawas B., Kalkan S., Liese J., Fruth K., Frerich B., Brieger J. (2015). Angiogenesis-related prognosis in patients with oral squamous cell carcinoma-role of the VEGF +936 C/T polymorphism. J. Oral Pathol. Med..

[54] Ko H.H., Lee J.J., Chen H.M., Kok S.H., Kuo M.Y.P., Cheng S.J., Chiang C.P. (2015). Upregulation of vascular endothelial growth factor mRNA level is significantly related to progression and prognosis of oral squamous cell carcinomas. J. Formos. Med. Assoc..

[55] Koukourakis M.I., Giatromanolaki A., Sivridis E., Gatter K.C., Harris A.L. (2013). High DLL4 expression in tumour-associated vessels predicts for favorable radiotherapy outcome in locally advanced squamous cell head-neck cancer (HNSCC). Angiogenesis.

[56] Lee S.Y., Chao-Nan Q., Seng O.A., Peiyi C., Bernice W.H., Swe M.S., Chii W.J., Jacqueline H.S., Chee S.K. (2012). Changes in specialized blood vessels in lymph nodes and their role in cancer metastasis. J. Transl. Med..

[57] Li C., Fan J., Song X., Zhang B., Chen Y., Li C., Mi K., Ma H., Song Y., Tao X. (2013). Expression of angiopoietin-2 and vascular endothelial growth factor receptor-3 correlates with lymphangiogenesis and angiogenesis and affects survival of oral squamous cell carcinoma. PLoS ONE.

[58] Marioni G., Staffieri A., Hagen R., Ottaviano G., Lionello M., Staffieri C., Giacomelli L., Blandamura S. (2013). Prognostic value of hypoxia-inducible factors (angiogenin and endoglin) in open partial laryngectomies: Uni- and multivariate analyses. Am. J. Otolaryngol..

[59] Marwah N., Parmar P., Parshad S., Yadav T., Gupta S., Sen R. (2016). Morphometric assessment of microvessel density in head and neck squamous cell carcinoma using immunomarker CD105 and its correlation with clinicopathological parameters. Clin. Cancer Investig. J..

[60] Matsui T., Shigeta T., Umeda M., Komori T. (2015). Vascular endothelial growth factor C (VEGF-C) expression predicts metastasis in tongue cancer. Oral Surg. Oral Med. Oral Pathol. Oral Radiol..

[61] Nair S., Nayak R., Bhat K., Kotrashetti V.S., Babji D. (2015). Immunohistochemical expression of CD105 and TGF-b1 in oral squamous cell carcinoma and adjacent apparently normal oral mucosa and its correlation with clinicopathologic features. Appl. Immunohistochem. Mol. Morphol..

[62] Nayak S., Goel M., Bhatia V., Chandra S., Makker A., Kumar S., Agrawal S.P., Mehrotra D., Rath S.K. (2013). Molecular and phenotypic expression of decorin as modulator of angiogenesis in human potentially malignant oral lesions and oral squamous cell carcinomas. Indian. J. Pathol. Microbiol..

[63] Sakata J., Hirosue A., Yoshida R., Kawahara K., Matsuoka Y., Yamamoto T., Nakamoto M., Hirayama M., Takahashi N., Nakamura T. (2019). HMGA2 contributes to distant metastasis and poor prognosis by promoting angiogenesis in oral squamous cell carcinoma. Int. J. Mol. Sci..

[64] Schlüter A., Weller P., Kanaan O., Nel I., Heusgen L., Höing B., Haßkamp P., Zander S., Mandapathil M., Dominas N. (2018). CD31 and VEGF are prognostic biomarkers in early-stage, but not in late-stage, laryngeal squamous cell carcinoma. BMC Cancer.

[65] Shi Y., Gong H.L., Zhou L., Tian J., Wang Y. (2014). Dickkopf-1 is a novel prognostic biomarker for laryngeal squamous cell carcinoma. Acta Otolaryngol..

[66] Supic G., Jovic N., Zeljic K., Kozomara R., Magic Z. (2012). Association of VEGF-A genetic polymorphisms with cancer risk and survival in advanced-stage oral squamous cell carcinoma patients. Oral Oncol..

[67] Uzun S., Korkmaz Y., Wuerdemann N., Arolt C., Puladi B., Siefer O.G., Dönmez H.G., Hufbauer M., Akgül B., Klussmann J.P. (2021). Comprehensive analysis of VEGFR2 expression in HPV-positive and -negative OPSCC reveals differing VEGFR2 expression patterns. Cancers.

[68] Xu H.M., Zhu J.G., Gu L., Hu S.Q., Wu H. (2016). VEGFR2 Expression in head and neck squamous cell carcinoma cancer cells mediates proliferation and invasion. Asian Pac. J. Cancer Prev..

[69] Yamagata Y., Tomioka H., Sakamoto K., Sato K., Harada H., Ikeda T., Kayamori K. (2017). CD163-positive macrophages within the tumor stroma are associated with lymphangiogenesis and lymph node metastasis in oral squamous cell carcinoma. J. Oral Maxillofac. Surg..

[70] Yanase M., Kato K., Yoshizawa K., Noguchi N., Kitahara H., Nakamura H. (2014). Prognostic value of vascular endothelial growth factors A and C in oral squamous cell carcinoma. J. Oral Pathol. Med..

[71] Agena S., Hirakawa H., Ikegami T., Kinjyo H., Kise N., Maeda H., Uezato J., Kondo S., Kiyuna A., Yamashita Y. (2021). Prognostic significance of hypoxia-inducible factor-1α expression in advanced pharyngeal cancer without human papillomavirus infection. J. Laryngol. Otol..

[72] Bernstein J.M., Andrews T.D., Slevin N.J., West C.M.L., Homer J.J. (2015). Prognostic value of hypoxia-associated markers in advanced larynx and hypopharynx squamous cell carcinoma: Hypoxia-Associated Biomarkers. Laryngoscope.

[73] Bharti A., Urs A., Kumar P. (2021). Significance of HIF-1α Expression and LOXL-2 Localization in progression of oral squamous cell carcinoma. Asian Pac. J. Cancer Prev..

[74] Choi H.G., Kim J.S., Kim K.H., Kim K.H., Sung M.W., Choe J.Y., Kim J.E., Jung Y.H. (2015). Expression of hypoxic signaling markers in head and neck squamous cell carcinoma and its clinical significance. Eur. Arch. Otorhinolaryngol..

[75] dos Santos M., Mercante A.M., Louro I.D., Gonçalves A.J., de Carvalho M.B., da Silva E.H., da Silva A.M. (2012). HIF1-alpha expression predicts survival of patients with squamous cell carcinoma of the oral cavity. PLoS ONE.

[76] Douglas C.M., Bernstein J.M., Ormston V.E., Hall R.C., Merve A., Swindell R., Valentine H.R., Slevin N.J., West C.M., Homer J.J. (2013). Lack of prognostic effect of carbonic anhydrase-9, hypoxia inducible factor-1α and Bcl-2 in 286 patients with early squamous cell carcinoma of the glottic larynx treated with radiotherapy. Clin. Oncol..

[77] Hong A., Zhang M., Veillard A.S., Jahanbani J., Lee C.S., Jones D., Harnett G., Clark J., Elliott M., Milross C. (2013). The prognostic significance of hypoxia inducing factor 1-α in oropharyngeal cancer in relation to human papillomavirus status. Oral Oncol..

[78] Kishimoto K., Yoshida S., Ibaragi S., Yoshioka N., Okui T., Hu G.F., Sasaki A. (2012). Hypoxia-induced up-regulation of angiogenin, besides VEGF, is related to progression of oral cancer. Oral Oncol..

[79] Li D., Zhou L., Jin B., Xie J., Dong P. (2013). Expression and significance of hypoxia-inducible factor–1α and survivin in laryngeal carcinoma tissue and cells. Otolaryngol. Neck Surg..

[80] Lim E., Kuo C.C., Tu H.F., Yang C.C. (2017). The prognosis outcome of oral squamous cell carcinoma using HIF-2α. J. Chin. Med. Assoc..

[81] Moreno-Galindo C., Hermsen M., García-Pedrero J.M., Fresno M.F., Suárez C., Rodrigo J.P. (2014). p27 and BCL2 expression predicts response to chemotherapy in head and neck squamous cell carcinomas. Oral Oncol..

[82] Mendes S.O., Santos M., Peterle G.T., de Lima Maia L., Stur E., Agostini L.P., de Carvalho M.B., Tajara E.H., Louro I.D., Trivilin L.O. (2014). HIF-1alpha expression profile in intratumoral and peritumoral inflammatory cells as a prognostic marker for squamous cell carcinoma of the oral cavity. PLoS ONE.

[83] Ou D., Garberis I., Adam J., Blanchard P., Nguyen F., Levy A., Casiraghi O., Gorphe P., Breuskin I., Janot F. (2018). Prognostic value of tissue necrosis, hypoxia-related markers and correlation with HPV status in head and neck cancer patients treated with bio- or chemo-radiotherapy. Radiother. Oncol..

[84] Pentheroudakis G., Nicolaou I., Kotoula V., Fountzilas E., Markou K., Eleftheraki A.G., Fragkoulidi A., Karasmanis I., Tsigka A., Angouridakis N. (2012). Prognostic utility of angiogenesis and hypoxia effectors in patients with operable squamous cell cancer of the larynx. Oral Oncol..

[85] Peterle G.T., Maia L.L., Trivilin L.O., de Oliveira M.M., Dos Santos J.G., Mendes S.O., Stur E., Agostini L.P., Rocha L.A., Moysés R.A. (2018). PAI-1, CAIX, and VEGFA expressions as prognosis markers in oral squamous cell carcinoma. J. Oral Pathol. Med..

[86] Siriwardena B.S.M.S., Karunathilaka H.D.N.U., Kumarasiri P.V.R., Tilakaratne W.M. (2020). Impact of histological and molecular parameters on prognosis of oral squamous cell carcinoma: Analysis of 290 cases. BioMed Res. Int..

[87] Swartz J.E., Wegner I., Noorlag R., van Kempen P.M.W., van Es R.J.J., de Bree R., Willems S.M.W. (2021). HIF-1a expression and differential effects on survival in patients with oral cavity, larynx, and oropharynx squamous cell carcinomas. Head Neck.

[88] Wachters J.E., Kop E., Slagter-Menkema L., Mastik M., van der Wal J.E., van der Vegt B., de Bock G.H., van der Laan B.F.A.M., Schuuring E. (2020). Distinct biomarker profiles and clinical characteristics in T1-T2 glottic and supraglottic carcinomas. Laryngoscope.

[89] Dalirsani Z., Memar B., Pakfetrat A., Mohtasham N., Anvari K., Kaveh S. (2020). Epidermal growth factor receptor expression in oral squamous cell carcinoma by immunohistochemical technique and its correlation with clinicopathological features. J. Kerman Univ. Med. Sci..

[90] Erkılınç G., Yasan H., Kumbul Y.Ç., Sivrice M.E., Durgun M. (2022). Expression of prostate-specific membrane antigen in the neovasculature of primary tumors and lymph node metastasis of laryngeal squamous cell carcinomas. J. Pathol. Transl. Med..

[91] Franz L., Tealdo G., Contro G., Bandolin L., Carraro V., Giacomelli L., Alessandrini L., Blandamura S., Marioni G. (2020). Biological tumor markers (maspin, CD105, NM23-H1) and disease relapse in laryngeal cancer: Cluster analysis. Head Neck.

[92] Guo Y.Q., Wang Q., Wang J.G., Gu Y.J., Song P.P., Wang S.Y., Qian X.Y., Gao X. (2022). METTL3 modulates m6A modification of CDC25B and promotes head and neck squamous cell carcinoma malignant progression. Exp. Hematol. Oncol..

[93] Haffner M.C., Laimer J., Chaux A., Schäfer G., Obrist P., Brunner A., Kronberger I.E., Laimer K., Gurel B., Koller J.B. (2012). High expression of prostate-specific membrane antigen in the tumor-associated neo-vasculature is associated with worse prognosis in squamous cell carcinoma of the oral cavity. Mod. Pathol..

[94] Ibrahim O.M., El-Ebiary H.A., Hegazy N.A., Fawaz S.A., El-Sharnouby M.M., Khafagy A.G. (2015). Does angiogenesis have a prognostic value in squamous cell carcinoma of the larynx?. Egypt J. Otolaryngol..

[95] Irani S., Dehghan A. (2018). The expression and functional significance of vascular endothelial-cadherin, CD44, and vimentin in oral squamous cell carcinoma. J. Int. Soc. Prev. Community Dent..

[96] Kawasaki G., Naruse T., Furukawa K., Umeda M. (2018). mTORC1 and mTORC2 expression levels in oral squamous cell carcinoma: An immunohistochemical and clinicopathological study. Anticancer Res..

[97] Li F., Liu Y., Kan X., Li Y., Liu M., Lu J.G. (2013). Elevated expression of integrin αv and β5 subunit in laryngeal squamous-cell carcinoma associated with lymphatic metastasis and angiogenesis. Pathol. Res. Pract..

[98] Li J.J., Mao X.H., Tian T., Wang W.M., Su T., Jiang C.H., Hu C.Y. (2019). Role of PFKFB3 and CD163 in oral squamous cell carcinoma angiogenesis. Curr. Med. Sci..

[99] Lin Y.T., Chuang H.C., Chen C.H., Armas G.L., Chen H.K., Fang F.M., Huang C.C., Chien C.Y. (2012). Clinical significance of erythropoietin receptor expression in oral squamous cell carcinoma. BMC Cancer.

[100] Lin N.N., Wang P., Zhao D., Zhang F.J., Yang K., Chen R. (2017). Significance of oral cancer-associated fibroblasts in angiogenesis, lymphangiogenesis, and tumor invasion in oral squamous cell carcinoma. J. Oral Pathol. Med..

[101] Maqsood A., Ali A., Zaffar Z., Mokeem S., Mokeem S.S., Ahmed N., Al-Hamoudi N., Vohra F., Javed F., Abduljabbar T. (2020). Expression of CD34 and α-SMA markers in oral squamous cell carcinoma differentiation. A histological and histo-chemical study. Int. J. Environ. Res. Public Health.

[102] Marioni G., Ottaviano G., Lovato A., Franz L., Bandolin L., Contro G., Giacomelli L., Alessandrini L., Stramare R., de Filippis C. (2020). Expression of maspin tumor suppressor and mTOR in laryngeal carcinoma. Am. J. Otolaryngol..

[103] Mariz B.A.L.A., Soares C.D., de Carvalho M.G.F., Jorge-Júnior J. (2019). FGF-2 and FGFR-1 might be independent prognostic factors in oral tongue squamous cell carcinoma. Histopathology.

[104] Mishev G., Deliverska E., Hlushchuk R., Velinov N., Aebersold D., Weinstein F., Djonov V. (2014). Prognostic value of matrix metalloproteinases in oral squamous cell carcinoma. Biotechnol. Equip..

[105] Nayak S., Goel M.M., Makker A., Bhatia V., Chandra S., Kumar S., Agarwal S.P. (2015). Fibroblast growth factor (FGF-2) and its receptors FGFR-2 and FGFR-3 may be putative biomarkers of malignant transformation of potentially malignant oral lesions into oral squamous cell carcinoma. PLoS ONE.

[106] Starska K., Forma E., Lewy-Trenda I., Stasikowska-Kanicka O., Skóra M., Bryś M. (2018). Fibroblast growth factor receptor 1 and 3 expression is associated with regulatory PI3K/AKT kinase activity, as well as invasion and prognosis, in human laryngeal cancer. Cell. Oncol..

[107] Stasikowska-Kanicka O., Wągrowska-Danilewicz M., Kulicka P., Danilewicz M. (2018). Overexpression of ADAM10 in oral squamous cell carcinoma with metastases. Pol. J. Pathol..

[108] Toyoda M., Kaira K., Shino M., Sakakura K., Takahashi K., Takayasu Y., Tominaga H., Oriuchi N., Nikkuni O., Suzuki M. (2015). CD98 as a novel prognostic indicator for patients with stage III/IV hypopharyngeal squamous cell carcinoma: CD98 expression in hypopharyngeal cancer. Head Neck.

[109] Troy J.D., Weissfeld J.L., Youk A.O., Thomas S., Wang L., Grandis J.R. (2013). Expression of EGFR, VEGF, and NOTCH1 suggest differences in tumor angiogenesis in HPV-positive and HPV-negative head and neck squamous cell carcinoma. Head Neck Pathol..

[110] Xia X., Du R., Zhao L., Sun W., Wang X. (2014). Expression of AEG-1 and microvessel density correlates with metastasis and prognosis of oral squamous cell carcinoma. Hum. Pathol..

[111] Yang X., Jin J., Yang J., Zhou L., Mi S., Qi G. (2021). Expression of Ubiquitin-specific protease 7 in oral squamous cell carcinoma promotes tumor cell proliferation and invasion. Genet. Mol. Biol..

[112] Zhou P.J., Yang Y.Y., Zhu Y.Q. (2020). Association between Endoglin and nm23-H1 Expression and the recurrence in elderly patients with laryngeal squamous cell carcinoma. Indian J. Pharm. Sci..

[113] Lionello M., Staffieri A., Marioni G. (2012). Potential prognostic and therapeutic role for angiogenesis markers in laryngeal carcinoma. Acta Otolaryngol..

[114] Byeon H.K., Ku M., Yang J. (2019). Beyond EGFR inhibition: Multilateral combat strategies to stop the progression of head and neck cancer. Exp. Mol. Med..

[115] Rehmani H.S., Issaeva N. (2020). EGFR in head and neck squamous cell carcinoma: Exploring possibilities of novel drug combinations. Ann. Transl. Med..

[116] Klein J.D., Grandis J.R. (2010). The molecular pathogenesis of head and neck cancer. Cancer Biol. Ther..

[117] Koole K., van Kempen P.M., Swartz J.E., Peeters T., van Diest P.J., Koole R., van Es R.J., Willems S.M. (2016). Fibroblast growth factor receptor 3 protein is overexpressed in oral and oropharyngeal squamous cell carcinoma. Cancer Med..

[118] Göke F., Bode M., Franzen A., Kirsten R., Goltz D., Göke A., Sharma R., Boehm D., Vogel W., Wagner P. (2013). Fibroblast growth factor receptor 1 amplification is a common event in squamous cell carcinoma of the head and neck. Mod. Pathol..

[119] Liu W., Bao Z.X., Shi L.J., Tang G.Y., Zhou Z.T. (2011). Malignant transformation of oral epithelial dysplasia: Clinicopathological risk factors and outcome analysis in a retrospective cohort of 138 cases. Histopathology.

[120] Castells M., Thibault B., Delord J.P., Couderc B. (2012). Implication of tumor microenvironment in chemoresistance: Tumor-associated stromal cells protect tumor cells from cell death. Int. J. Mol. Sci..

[121] Garín M.I., Chu C.C., Golshayan D., Cernuda-Morollón E., Wait R., Lechler R.I. (2007). Galectin-1: A key effector of regulation mediated by CD4+CD25+ T cells. Blood.

[122] Cedeno-Laurent F., Opperman M., Barthel S.R., Kuchroo V.K., Dimitroff C.J. (2012). Galectin-1 triggers an immunoregulatory signature in Th cells functionally defined by IL-10 expression. J. Immunol..

[123] Rubinstein N., Alvarez M., Zwirner N.W., Toscano M.A., Ilarregui J.M., Bravo A. (2004). Targeted inhibition of galectin-1 gene expression in tumor cells results in heightened T cell-mediated rejection: A potential mechanism of tumor-immune privilege. Cancer Cell..

[124] Sharma P.S., Sharma R., Tyagi T. (2011). VEGF/VEGFR pathway inhibitors as anti-angiogenic agents: Present and future. Curr. Cancer Drug Targets.

[125] Marioni G., Giacomelli L., D’Alessandro E., Staffieri C., Guzzardo V., Staffieri A., Blandamura S. (2008). Laryngeal carcinoma recurrence rate and disease-free interval are related to CD105 expression but not to vascular endothelial growth factor 2 (Flk-1/Kdr) expression. Anticancer Res..

[126] Tanaka F., Ishikawa S., Yanagihara K., Miyahara R., Kawano Y., Li M., Otake Y., Wada H. (2002). Expression of angiopoietins and its clinical significance in non-small cell lung cancer. Cancer Res..

[127] Fei J., Hong A., Dobbins T.A., Jones D., Lee C.S., Loo C., Al-Ghamdi M., Harnett G.B., Clark J., O’Brien C.J. (2009). Prognostic significance of vascular endothelial growth factor in squamous cell carcinomas of the tonsil in relation to human papillomavirus status and epidermal growth factor receptor. Ann. Surg. Oncol..

[128] Hayashi Y., Yokota A., Harada H., Huang G. (2019). Hypoxia/pseudohypoxia-mediated activation of hypoxia-inducible factor-1α in cancer. Cancer Sci..

[129] Maynard M.A., Ohh M. (2007). The role of hypoxia-inducible factors in cancer. Cell. Mol. Life. Sci..

[130] Pouysségur J., Dayan F., Mazure N.M. (2006). Hypoxia signalling in cancer and approaches to enforce tumour regression. Nature.

[131] Fillies T., Werkmeister R., van Diest P.J., Brandt B., Joos U., Buerger H. (2005). HIF1-alpha overexpression indicates a good prognosis in early stage squamous cell carcinomas of the oral floor. BMC. Cancer.

[132] Zhou J., Huang S., Wang L., Yuan X., Dong Q., Zhang D., Wang X. (2017). Clinical and prognostic significance of HIF-1α lpha overexpression in oral squamous cell carcinoma: A meta-analysis. World J. Surg. Oncol..

[133] Yang S., Xiao H., Cao L. (2021). Recent advances in heat shock proteins in cancer diagnosis, prognosis, metabolism and treatment. Biomed. Pharmacother..

[134] Loncaster J.A., Harris A.L., Davidson S.E., Logue J.P., Hunter R.D., Wycoff C.C., Pastorek J., Ratcliffe P.J., Stratford I.J., West C.M. (2001). Carbonic anhydrase (CA IX) expression, a potential new intrinsic marker of hypoxia: Correlations with tumor oxygen measurements and prognosis in locally advanced carcinoma of the cervix. Cancer Res..

[135] Liu L., Li M., Zhang C., Zhang J., Li G., Zhang Z., He X., Fan M. (2018). Prognostic value and clinicopathologic significance of nm23 in various cancers: A systematic review and meta-analysis. Int. J. Surg..

[136] Marshall J.C., Collins J., Marino N., Steeg P. (2010). The Nm23-H1 metastasis suppressor as a translational target. Eur. J. Cancer.

[137] Heimann R., Ferguson D.J., Hellman S. (1998). The relationship between nm23, angiogenesis, and the metastatic proclivity of node-negative breast cancer. Cancer Res..

[138] Youn B., Kim H.D., Kim J. (2008). Nm23-H1/nucleoside diphosphate kinase as a key molecule in breast tumor angiogenesis. Expert. Opin. Ther. Targets.

[139] Kim J.H., Cho N.Y., Bae J.M., Kim K.J., Rhee Y.Y., Lee H.S., Kang G.H. (2015). Nuclear maspin expression correlates with the CpG island methylator phenotype and tumor aggressiveness in colorectal cancer. Int. J. Clin. Exp. Pathol..

[140] Reina J., Zhou L., Fontes M.R.M., Panté N., Cella N. (2019). Identification of a putative nuclear localization signal in the tumor suppressor maspin sheds light on its nuclear import regulation. FEBS Open Bio.

[141] Marioni G., Giacomelli L., D’Alessandro E., Marchese-Ragona R., Staffieri C., Ferraro S.M., Staffieri A., Blandamura S. (2008). Nuclear localization of mammary serine protease inhibitor (MASPIN): Is its impact on the prognosis in laryngeal carcinoma due to a proapoptotic effect?. Am. J. Otolaryngol..

[142] Lovato A., Franz L., Carraro V., Bandolin L., Contro G., Ottaviano G., de Filippis C., Blandamura S., Alessandrini L., Marioni G. (2020). Maspin expression and anti-apoptotic pathway regulation by bcl2 in laryngeal cancer. Ann. Diagn. Pathol..

[143] Zhong Y., Lu Y.T., Sun Y., Shi Z.H., Li N.G., Tang Y.P., Duan J.A. (2018). Recent opportunities in matrix metalloproteinase inhibitor drug design for cancer. Expert. Opin. Drug Discov..

[144] Coussens L.M., Fingleton B., Matrisian L.M. (2002). Matrix metalloproteinase inhibitors and cancer: Trials and tribulations. Science.

[145] Egeblad M., Werb Z. (2002). New functions for the matrix metalloproteinases in cancer progression. Nat. Rev. Cancer.

[146] Quintero-Fabián S., Arreola R., Becerril-Villanueva E., Torres-Romero J.C., Arana-Argáez V., Lara-Riegos J., Ramírez-Camacho M.A., Alvarez-Sánchez M.E. (2019). Role of matrix metalloproteinases in angiogenesis and cancer. Front. Oncol..

[147] Gumina R.J., Kirschbaum N.E., Rao P.N., van Tuinen P., Newman P.J. (1996). The human PECAM1 gene maps to 17q23. Genomics.

[148] Dehne N., Brüne B. (2009). HIF-1 in the inflammatory microenvironment. Exp. Cell Res..

[149] Sitkovsky M., Lukashev D. (2005). Regulation of immune cells by local-tissue oxygen tension: HIF1 alpha and adenosine receptors. Nat. Rev. Immunol..

[150] Brandwein-Gensler M., Teixeira M.S., Lewis C.M., Lee B., Rolnitzky L., Hille J.J., Genden E., Urken M.L., Wang B.Y. (2005). Oral squamous cell carcinoma: Histologic risk assessment, but not margin status, is strongly predictive of local disease-free and overall survival. Am. J. Surg. Pathol..

[151] Marioni G., Marino F., Blandamura S., D’Alessandro E., Giacomelli L., Guzzardo V., Lionello M., De Filippis C., Staffieri A. (2010). Neoangiogenesis in laryngeal carcinoma: Angiogenin and CD105 expression is related to carcinoma recurrence rate and disease-free survival. Histopathology.

[152] Talks K.L., Turley H., Gatter K.C., Maxwell P.H., Pugh C.W., Ratcliffe P.J., Harris A.L. (2000). The expression and distribution of the hypoxia-inducible factors HIF-1alpha and HIF-2alpha in normal human tissues, cancers, and tumor-associated macrophages. Am. J. Pathol..

[153] Placencio V.R., DeClerck Y.A. (2015). Plasminogen activator inhibitor-1 in cancer: Rationale and insight for future therapeutic testing. Cancer Res..

[154] Rupertus K., Sinistra J., Scheuer C., Nickels R.M., Schilling M.K., Menger M.D., Kollmar O. (2014). Interaction of the chemokines I-TAC (CXCL11) and SDF-1 (CXCL12) in the regulation of tumor angiogenesis of colorectal cancer. Clin. Exp. Metastasis.

[155] Yamada K., Maishi N., Akiyama K., Towfik Alam M., Ohga N., Kawamoto T., Shindoh M., Takahashi N., Kamiyama T., Hida Y. (2015). CXCL12–CXCR7 axis is important for tumor endothelial cell angiogenic property. Int. J. Cancer.

[156] Sun X., Cheng G., Hao M., Zheng J., Zhou X., Zhang J., Taichman R.S., Pienta K.J., Wang J. (2010). CXCL12 / CXCR4 / CXCR7 chemokine axis and cancer progression. Cancer Metastasis Rev..

[157] Watanabe K., Penfold M.E., Matsuda A., Ohyanagi N., Kaneko K., Miyabe Y., Matsumoto K., Schall T.J., Miyasaka N., Nanki T. (2010). Pathogenic role of CXCR7 in rheumatoid arthritis. Arthritis Rheum..

[158] Goerdt S., Bhardwaj R., Sorg C. (1993). Inducible expression of MS-1 high-molecular-weight protein by endothelial cells of continuous origin and by dendritic cells/macrophages in vivo and in vitro. Am. J. Pathol..

[159] Cho J.H., Kim H.S., Park C.S., Kim J.K., Jung K.Y., Shin B.K., Kim H.K. (2007). Maspin expression in early oral tongue cancer and its relation to expression of mutant-type p53 and vascular endothelial growth factor (VEGF). Oral Oncol..

[160] Impola U., Uitto V.J., Hietanen J., Hakkinen L., Zhang L., Larjava H., Isaka K., Saarialho-Kere U. (2004). Differential expression of matrilysin-1 (MMP-7), 92 kD gelatinase (MMP-9), and metalloelastase (MMP-12) in oral verrucous and squamous cell cancer. J. Pathol..

[161] Conway R.E., Petrovic N., Li Z., Heston W., Wu D., Shapiro L.H. (2006). Prostate-specific membrane antigen regulates angiogenesis by modulating integrin signal transduction. Mol. Cell. Biol..

[162] Cirri P., Chiarugi P. (2011). Cancer associated fibroblasts: The dark side of the coin. Am. J. Cancer Res..

[163] Roholl P.J., Elbers H.R., Prinsen I., Claessens J.A., van Unnik J.A. (1990). Distribution of actin isoforms in sarcomas: An immunohistochemical study. Hum. Pathol..

[164] Wachters J.E., Schrijvers M.L., Slagter-Menkema L., Mastik M., de Bock G.H., Langendijk J.A., Kluin P.M., Schuuring E., van der Laan B.F., van der Wal J.E. (2013). Prognostic significance of HIF-1a, CA-IX, and OPN in T1-T2 laryngeal carcinoma treated with radiotherapy: Hypoxia markers in laryngeal cancer. Laryngoscope.

[165] Franz L., Alessandrini L., Ottaviano G., di Carlo R., Fasanaro E., Ramacciotti G., Contro G., Marioni G. (2020). Postoperative radiotherapy for laryngeal cancer. The prognostic role of programmed death-ligand 1: An immune microenvironment-based cluster analysis. Pathol. Res. Pract..

[166] Franz L., Alessandrini L., Calvanese L., Crosetta G., Frigo A.C., Marioni G. (2021). Angiogenesis, programmed death ligand 1 (PD-L1) and immune microenvironment association in laryngeal carcinoma. Pathology.

[167] Taube J.M., Anders R.A., Young G.D. (2012). Colocalization of inflammatory response with B7-H1 expression in human melanocytic lesions supports an adaptive resistance mechanism of immune escape. Sci. Transl. Med..

[168] Marioni G., D’Alessandro E., Giacomelli L., Staffieri A. (2010). CD105 is a marker of tumour vasculature and a potential target for the treatment of head and neck squamous cell carcinoma. J. Oral Pathol. Med..

[169] Marioni G., Franz L., Ottaviano G., Contro G., Tealdo G., Carli A., Frigo A.C., Nicolai P., Alessandrini L. (2020). Prognostic significance of CD105- and CD31-assessed microvessel density in paired biopsies and surgical samples of laryngeal carcinoma. Cancers.

[170] Marioni G., Ottaviano G., Marchese-Ragona R., Fasanaro E., Tealdo G., Zanotti C., Randon B., Giacomelli L., Stellini E., Blandamura S. (2017). Nuclear survivin expression correlates with endoglin-assessed microvascularisation in laryngeal carcinoma. J. Clin. Pathol..

[171] Kramer B., Polit M., Birk R., Rotter N., Aderhold C. (2018). HIF-1α and mTOR-Possible Novel Strategies of Targeted Therapies in p16-positive and -negative HNSCC. Cancer Genom. Proteom..

[172] Lionello M., Lovato A., Staffieri A., Blandamura S., Turato C., Giacomelli L., Staffieri C., Marioni G. (2014). The EGFR-mTOR pathway and laryngeal cancer angiogenesis. Eur. Arch. Otorhinolaryngol..

[173] Alessandrini L., Ferrari M., Taboni S., Sbaraglia M., Franz L., Saccardo T., Del Forno B.M., Agugiaro F., Frigo A.C., Dei Tos A.P. (2022). Tumor-stroma ratio, neoangiogenesis and prognosis in laryngeal carcinoma. A pilot study on preoperative biopsies and matched surgical specimens. Oral Oncol..

[174] Marioni G., D’Alessandro E., Giacomelli L., De Filippis C., Calgaro N., Sari M., Staffieri A., Blandamura S. (2006). Maspin nuclear localization is related to reduced density of tumour-associated micro-vessels in laryngeal carcinoma. Anticancer Res..

[175] Chaturvedi A.K., Engels E.A., Pfeiffer R.M., Hernandez B.Y., Xiao W., Kim E., Jiang B., Goodman M.T., Sibug-Saber M., Cozen W. (2023). Human Papillomavirus and Rising Oropharyngeal Cancer Incidence in the United States. J. Clin. Oncol..

[176] Hong A.M., Dobbins T.A., Lee C.S., Jones D., Harnett G.B., Armstrong B.K., Clark J.R., Milross C.G., Kim J., O’Brien C.J. (2010). Human papillomavirus predicts outcome in oropharyngeal cancer in patients treated primarily with surgery or radiation therapy. Br. J. Cancer.

[177] Li R., Xiao L., Gong T., Liu J., Li Y., Zhou X., Li Y., Zheng X. (2023). Role of oral microbiome in oral oncogenesis, tumor progression, and metastasis. Mol. Oral Microbiol..

[178] Muzio L.L., Ballini A., Cantore S., Bottalico L., Charitos I.A., Ambrosino M., Nocini R., Malcangi A., Dioguardi M., Cazzolla A.P. (2021). Overview of Candida albicans and Human Papillomavirus (HPV) Infection Agents and their Biomolecular Mechanisms in Promoting Oral Cancer in Pediatric Patients. Biomed Res. Int..

[179] Stokes W.A., Molina E., McDermott J.D., Morgan R.L., Bickett T., Fakhoury K.R., Amini A., Karam S.D. (2021). Survival impact of angiotensin-converting enzyme inhibitors and angiotensin II receptor antagonists in head and neck cancer. Head Neck.

[180] Kwok-Shing W.M., Ando H., Ukena K., Nagata S. (2021). Subchapter 42B-Angiotensin II. Handbook of Hormones.

[181] Chang Q., Bournazou E., Sansone P., Berishaj M., Gao S.P., Daly L., Wels J., Theilen T., Granitto S., Zhang X. (2013). The IL-6/JAK/Stat3 feed-forward loop drives tumorigenesis and metastasis. Neoplasia.

[182] Hu Y., Lu L., Qiu Z., Huang Q., Chen Y., Chen L. (2018). Mechanical stretch aggravates aortic dissection by regulating MAPK pathway and the expression of MMP-9 and inflammation factors. Biomed. Pharmacother..

[183] Zhang H.Y., Zheng X.Z., Wang X.H., Xuan X.Y., Wang F., Li S.S. (2012). S100A4 mediated cell invasion and metastasis of esophageal squamous cell carcinoma via the regulation of MMP-2 and E-cadherin activity. Mol. Biol. Rep..

[184] Kasza A. (2013). IL-1 and EGF regulate expression of genes important in inflammation and cancer. Cytokine.

[185] Paik J.S., Cho W.K., Oh E.H., Lee S.B., Yang S.W. (2012). Palmitate induced secretion of IL-6 and MCP-1 in orbital fibroblasts derived from patients with thyroid-associated ophthalmopathy. Mol. Vis..

[186] Cui G., Yuan A., Sun Z., Zheng W., Pang Z. (2018). IL-1β/IL-6 network in the tumor microenvironment of human colorectal cancer. Pathol. Res. Pract..

[187] Lu S.L., Reh D., Li A.G., Woods J., Corless C.L., Kulesz Martin M., Wang X.J. (2004). Overexpression of transforming growth factor beta1 in head and neck epithelia results in inflammation, angiogenesis and epithelial hyperproliferation. Cancer Res..

[188] Biobe G.C., Schiemann W.P., Lodish H.E. (2000). Role of transforming growth factor beta in human disease. N. Engl. J. Med..

[189] Vlahakis S.R., Villasis-Keever A., Gomez T.S., Bren G.D., Paya C.V. (2003). Human immunodeficiency virus-induced apoptosis of human hepatocytes via CXCR4. J. Infect. Dis..

[190] Puchert M., Obst J., Koch C., Zieger K., Engele J. (2020). CXCL11 promotes tumor progression by the biased use of the chemokine receptors CXCR3 and CXCR7. Cytokine.

[191] Dienstmann R., Rodon J., Prat A., Perez-Garcia J., Adamo B., Felip E., Cortes J., Iafrate A.J., Nuciforo P., Tabernero J. (2014). Genomic aberrations in the FGFR pathway: Opportunities for targeted therapies in solid tumors. Ann. Oncol..

[192] Hierro C., Rodon J., Tabernero J. (2015). Fibroblast Growth Factor (FGF) Receptor/FGF inhibitors: Novel targets and strategies for optimization of response of solid tumors. Semin. Oncol..

[193] Swartz J.E., Pothen A.J., van Kempen P.M.W., Stegeman I., Formsma F.K., Cann E.M., Willems S.M., Grolman W. (2016). Poor prognosis in human papillomavirus-positive oropharyngeal squamous cell carcinomas that overexpress hypoxia inducible factor-1α: Hypoxia inducible factor-1α as predictor of poor prognosis in HPV-positive propharyngeal SCC. Head Neck.

[194] Masterson L., Moualed D., Liu Z.W., Howard J.E., Dwivedi R.C., Tysome J.R., Benson R., Sterling J.C., Sudhoff H., Jani P. (2014). De-escalation treatment protocols for human papillomavirus-associated oropharyngeal squamous cell carcinoma: A systematic review and meta-analysis of current clinical trials. Eur. J. Cancer.

[195] Nichols A.C., Lang P., Prisman E., Berthelet E., Tran E., Hamilton S., Wu J., Fung K., de Almeida J.R., Bayley A. (2020). Treatment de-escalation for HPV-associated oropharyngeal squamous cell carcinoma with radiotherapy vs. trans-oral surgery (ORATOR2): Study protocol for a randomized phase II trial. BMC Cancer.

[196] Begg A.C., Stewart F.A., Vens C. (2011). Strategies to improve radiotherapy with targeted drugs. Nat. Rev. Cancer.

[197] Bonner J.A., Harari P.M., Giralt J., Cohen R.B., Jones C.U., Sur R.K., Raben D., Baselga J., Spencer S.A., Zhu J. (2010). Radiotherapy plus cetuximab for locoregionally advanced head and neck cancer: 5-year survival data from a phase 3 randomised trial, and relation between cetuximab-induced rash and survival. Lancet Oncol..

[198] Harari P.M., Harris J., Kies M.S., Myers J.N., Jordan R.C., Gillison M.L., Foote R.L., Machtay M., Rotman M., Khuntia D. (2014). Postoperative chemoradiotherapy and cetuximab for high-risk squamous cell carcinoma of the head and neck: Radiation Therapy Oncology Group RTOG-0234. J. Clin. Oncol..

[199] National Comprehensive Cancer Network NCCN Guidelines for Head and Neck Cancers, Version 1. https://www.nccn.org/professionals/physician_gls/pdf/head-and-neck.pdf.

[200] Vermorken J.B., Mesia R., Rivera F., Remenar E., Kawecki A., Rottey S., Erfan J., Zabolotnyy D., Kienzer H.R., Cupissol D. (2008). Platinum-based chemotherapy plus cetuximab in head and neck cancer. N. Engl. J. Med..

[201] Burtness B., Goldwasser M.A., Flood W., Mattar B., Forastiere A.A. (2005). Eastern Cooperative Oncology Group. Phase III randomized trial of cisplatin plus placebo compared with cisplatin plus cetuximab in metastatic/recurrent head and neck cancer: An Eastern Cooperative Oncology Group study. J. Clin. Oncol..

[202] Adkins D., Mehan P., Ley J., Siegel M.J., Siegel B.A., Dehdashti F., Jiang X., Salama N.N., Trinkaus K., Oppelt P. (2018). Pazopanib plus cetuximab in recurrent or metastatic head and neck squamous cell carcinoma: An open-label, phase 1b and expansion study. Lancet Oncol..

[203] Hyytiäinen A., Wahbi W., Väyrynen O., Saarilahti K., Karihtala P., Salo T., Al-Samadi A. (2021). Angiogenesis inhibitors for head and neck squamous cell carcinoma treatment: Is there still hope?. Front. Oncol..

[204] Micaily I., Johnson J., Argiris A. (2020). An update on angiogenesis targeting in head and neck squamous cell carcinoma. Cancers Head Neck.

[205] Saada-Bouzid E., Le Tourneau C. (2019). Beyond EGFR Targeting in SCCHN: Angiogenesis, PI3K, and other molecular targets. Front. Oncol..

[206] Argiris A., Kotsakis A.P., Hoang T., Worden F.P., Savvides P., Gibson M.K., Gyanchandani R., Blumenschein G.R., Chen H.X., Grandis J.R. (2013). Cetuximab and bevacizumab: Preclinical data and phase II trial in recurrent or metastatic squamous cell carcinoma of the head and neck. Ann. Oncol..

[207] Argiris A., Li S., Savvides P., Ohr J.P., Gilbert J., Levine M.A., Chakravarti A., Haigentz M., Saba N.F., Ikpeazu C.V. (2019). Phase III randomized trial of chemotherapy with or without bevacizumab in patients with recurrent or metastatic head and neck cancer. J. Clin. Oncol..

[208] Wachsberger P., Burd R., Dicker A.P. (2003). Tumor response to ionizing radiation combined with antiangiogenesis or vascular targeting agents: Exploring mechanisms of interaction. Clin. Cancer Res..

[209] Argiris A., Bauman J.E., Ohr J., Gooding W.E., Heron D.E., Duvvuri U., Kubicek G.J., Posluszny D.M., Vassilakopoulou M., Kim S. (2016). Phase II randomized trial of radiation therapy, cetuximab, and pemetrexed with or without bevacizumab in patients with locally advanced head and neck cancer. Ann. Oncol..

[210] Yao M., Galanopoulos N., Lavertu P., Fu P., Gibson M., Argiris A., Rezaee R., Zender C., Wasman J., Machtay M. (2015). Phase II study of bevacizumab in combination with docetaxel and radiation in locally advanced squamous cell carcinoma of the head and neck. Head Neck.

[211] Hsu H.W., Wall N.R., Hsueh C.T., Kim S., Ferris R.L., Chen C.S., Mirshahidi S. (2014). Combination antiangiogenic therapy and radiation in head and neck cancers. Oral Oncol..

[212] Choong N.W., Kozloff M., Taber D., Hu H.S., Wade J., Ivy P., Karrison T.G., Dekker A., Vokes E.E., Cohen E.E. (2010). Phase II study of sunitinib malate in head and neck squamous cell carcinoma. Invest. New Drugs.

[213] Chen T.H., Chang P.M., Yang M.H. (2021). Combination of pembrolizumab and lenvatinib is a potential treatment option for heavily pretreated recurrent and metastatic head and neck cancer. J. Chin. Med. Assoc..

[214] Ollauri-Ibáñez C., Ayuso-Íñigo B., Pericacho M. (2021). Hot and Cold Tumors: Is Endoglin (CD105) a Potential Target for Vessel Normalization?. Cancers.

[215] Apolo A.B., Karzai F.H., Trepel J.B., Alarcon S., Lee S., Lee M.-J., Tomita Y., Cao L., Yu Y., Merino M.J. (2017). A Phase II Clinical Trial of TRC105 (Anti-Endoglin Antibody) in Adults with Advanced/Metastatic Urothelial Carcinoma. Clin. Genitourin. Cancer.

[216] Paauwe M., Schoonderwoerd M.J.A., Helderman R.F.C.P., Harryvan T.J., Groenewoud A., Van Pelt G.W., Bor R., Hemmer D.M., Versteeg H.H., Ewa Snaar-Jagalska B. (2018). Endoglin expression on cancer-associated fibroblasts regulates invasion and stimulates colorectal cancer metastasis. Clin. Cancer Res..

[217] Paauwe M., Heijkants R.C., Oudt C.H., Van Pelt G.W., Cui C., Theuer C.P., Hardwick J.C.H., Sier C.F.M., Hawinkels L.J.A.C. (2016). Endoglin targeting inhibits tumor angiogenesis and metastatic spread in breast cancer. Oncogene.

[218] Karzai F.H., Apolo A.B., Cao L., Madan R.A., Adelberg D.E., Parnes H., McLeod D.G., Harold N., Peer C., Yu Y. (2015). A phase i study of TRC105 anti-endoglin (CD105) antibody in metastatic castration-resistant prostate cancer. BJU Int..

[219] ClinicalTrials.gov Identifiers: NCT04770896. NCT04770896.

[220] ClinicalTrials.gov Identifiers: NCT04712643. NCT04712643.

[221] ClinicalTrials.gov Identifiers: NCT04732286. NCT04732286.

[222] Huang Y.H., Goel S., Duda D.G., Fukumura D., Jain R.K. (2013). Vascular normalization as an Emerging strategy to enhance cancer immunotherapy. Cancer Res..

[223] Polverini P.J., D’Silva N.J., Lei Y.L. (2018). Precision therapy of head and neck squamous cell carcinoma. J. Dent. Res..

