# Supplementary material for: Diagnostic, Prognostic, and Therapeutic Role for Angiogenesis Markers in Head and Neck Squamous Cell Carcinoma: A Narrative Review
Source: Int J Mol Sci. 2023 Jun 27;24(13):10733. doi: 10.3390/ijms241310733 (PMC10341715; doi:10.3390/ijms241310733)
Supplement: Supplementary file 1 [file ijms-24-10733-s001.zip › ijms-2388488-supplementary.pdf]

Table S1. Angiogenesis in HNSCC. Diagnostic markers.

Author	Year	Country	Marker Studied	Method of detection	Tumor type	No. of cases	Mean Age (Range)	Sex (M/F)	Remarks
Aggarwal et al. [41]	2014	India	VEGF	qRT-PCR, WB, ELISA	OSCC Oropharyngeal SCC	70	42.93 ± 12.25 (24-69)	60/10	- Serum VEGF levels showed an increasing trend with clinical stage and lymph node involvement.
Aggarwal et al. [31]	2015	India	Galectin-1 Galectin-3	qRT-PCR WB IHC serum	OSCC	60	46.5 ± 10.9 (24-80)	51/9	- Serum levels of gal-1 and gal-3 may serve as plausible markers for oral squamous cell carcinoma and may be useful in screening population at a higher risk.
Etemad-Moghadam et al. [49]	2019	Iran	ADAM10 EGFR CD105	IHC	OSCC	50	NR (19-88)	28/20	- A significant negative correlation was found between ADAM10 and CD105.
Kayamori et al. [36]	2016	Japan	NOTCH3	IHC WB RT-PCR	OSCC	64	NR (29-79)	49/15	-OSCCs stimulated the surrounding CAFs to express NOTCH3 in a cell-to-cell contact dependent manner, which activated the CAFs and resulted in their involvement in angiogenesis, thereby promoting tumor growth.
Mariz et al. [104]	2019	Brazil	FGF-2 FGFR-1	IHC	OSCC	85	55.5 (19-89)	46/39	-FGF-2 overexpression and FGFR-1 overexpression were correlated with metastasis and a worse prognosis in OSCC and could be used independently to predict the prognosis of patients.
Nayak et al. [106]	2015	India	FGF-2 FGFR-2 FGFR-3	IHC qRT-PCR	Potentially malignant oral lesions, OSCC	232	42 (8-85)	172/60	-Upregulation of FGF-2, FGFR-2 and FGFR-3 expression both at phenotypic and molecular level from PMOL to OSCC with statistically significant co-expression of FGF-2 and FGF-2.<br>-FGF-2 and FGFR-2 expression may serve as an adjunct to histopathologic assessment of epithelial dysplasia for evaluating progression and malignant transformation in PMOLs.
Tokmak et al. [38]	2021	Turkey	Galectin-3	IHC	OSCC	60	64.1 (34-93)	39/21	-Nuclear galectin-3 staining was not found to be associated with OS or recurrence rates<br>-Total expression score of galectin-3 was found to be significantly higher in high-grade tumors than low-grade ones.

Troy et al. [110]	2013	USA	EGFR NOTCH1 VEGF	IHC	HNSCC	67	NR (18–79)	50/17	-HPV-negative HNSCC over-expressed EGFR relative to HPV-positive HNSCC.<br>-VEGF and NOTCH1 were unrelated to HPV status.<br>-EGFR was associated with VEGF in HPV-negative but not HPV-positive HNSCC.<br>-NOTCH1 and VEGF were associated in HPV-negative but not HPV positive tumors.
Uzun et al. [68]	2021	Germany	VEGFR2	IHC	Oropharyngeal SCC	56	60.2	43/13	-HPV-infection induced significant downregulation of VEGFR2 in cancer cells compared to HPV-negative tumor cells.<br>-The intensity of VEGFR2 staining differed only in HPV-positive oropharyngeal SCC and was upregulated in the blood vessels of tumor-containing regions.
Xu et al. [69]	2016	China	VEGFR2	WB IHC	HNSCC	109	NR	NR	-Knockdown of VEGFR2 in Hep2 cells could arrest the cell cycle in G0/G1, leading to a decrease in proliferation.<br>-MAPK/ERK signal pathways and expression of CDK1 downstream of VEGFR2 might regulate proliferation and cell cycle arrest.<br>-Down-regulate VEGRF2 in Hep2 cells could significantly affect the invasion ability.
Yang et al. [40]	2013	Sweden	COX-2 CD44v6 CD147	IHC	Hypo-pharyngeal SCC	101	61.15.00	99/2	-The COX-2 and CD147 were significantly increased in carcinoma tissues compared to the epithelium adjacent to carcinoma.<br>-The expression of COX-2, CD44v6, and CD147 were significantly associated with T classification, lymph node metastasis and clinical stage.
Yang et al. [112]	2021	China	USP7 Ki-67 MMP2 VEGF	IHC WB	OSCC	92	70 cases ≥ 50 yrs 22 cases < 50 yrs	66/26	-USP7 is weakly expressed in normal oral mucosa tissues but with high expression in OSCC tissues.<br>-USP7 expression is positively associated to migration and invasion-related factors MMP2, MMP9 and VEGF.<br>-USP7 promotes cell proliferation, inhibit apoptosis, enhances cell migration and invasion, activates the Akt/ERK pathway.

Abbreviations ADAM10: a disintegrin and metalloproteinase-10, CAFs: Cancer-associated fibroblasts, CDK1: cyclin-dependent kinase 1, COX-2: cyclooxygenase-2, EGFR: Epidermal growth factor receptor, ELISA: enzyme-linked immunosorbent assay, FGF-2: Fibroblast growth factor-2, FGFR-1: fibroblast growth factor receptor-1, Gal-1: galectin-1, Gal-3: galectin-3, HNSCC: head and neck squamous cell carcinoma, HPV: human papilloma virus, IHC: Immunohistochemistry, MAPK/ERK: mitogen-activated protein kinase/extracellular signal-regulated kinase MMP-9: Matrix metalloproteinase-9, NOTCH3: Neurogenic locus notch homolog protein 3, NR: not reported, OS: overall survival, OSCC: oral squamous cell carcinoma, PMOLs: potentially malignant oral lesions, qRT-PCR: quantitative real time protein chain reaction, SCC: squamous cell carcinoma, USP7: Ubiquitin-specific protease 7, VEGF: vascular endothelial growth factor, VEGFR-3,2: vascular endothelial growth factor-3,2, WB: Western blot.

Table S2. Angiogenesis in HNSCC. Prognostic markers

Author	Year	Country	Marker Studied	Method of detection	Tumor type	No. of cases	Mean Age (Range)	Sex (M/F)	Remarks
Agena et al. [72]	2021	Japan	HIF-1α<br>GLUT-1	IHC	Oro-pharyngeal SCC<br>Hypo-pharyngeal SCC	80	66 (median) (39–82)	69/11	-HIF-1α expression was an independent risk factor for poor prognosis for advanced human papillomavirus-unrelated pharyngeal cancer.
Al-Shareef e t al. [42]	2016	Japan	VEGF-C, -D<br>VEGFR-3<br>CCR7<br>NRP1, NRP2<br>SEMA3E	IHC	Oral tongue SCC	80	NR (22-92)	55/25	- Significant association between lymph node metastasis and the expression levels of VEGF-C, VEGFR-3, CCR7, NRP1, and SEMA3E.
Ansari et al. [43]	2020	Pakistan	CD117<br>CD34	IHC	OSCC	60	60.10 ± 16.01	40/20	- Increased MVD in poorly differentiated OSCC as compared to well and moderately differentiated OSCC suggested that it could be used as an additional criterion to histologically grade the tumors.
Bernstein et al. [73]	2015	UK	CA-IX<br>HIF-1α	IHC	LSCC<br>Hypo-pharyngeal SCC	114	63 (37-86)	86/28	-CA-IX expression is an adverse prognostic factor for DSS.<br>-CA-IX expression may confer a more aggressive tumor phenotype.
Bertini et al. [44]	2016	Brazil	VEGF<br>VEGF-C<br>CD105<br>D2-40	IHC	OSCC	59	59.2	45/14	- High MVD values occurred in cases with greater cell proliferation. No relationship was determined between the growth factors VEGF and VEGF-C and MVD and lymphatic density, respectively.
Bharti et al. [74]	2020	India	HIF-1α<br>LOXL-2	IHC	OSCC	90	49.5 (12-77)	75/15	-Alteration in the immunoexpression of LOXL-2 from nuclear to cytoplasmic and HIF-1α immunoexpression might be an important factor in progression of OSCC.

Chen et al. [45]	2016	Taiwan	CD105	ELISA<br>qRT-PCR<br>IHC	OSCC	71	51.7 (34–82)	68/3	- Patients with higher peripheral vein CD105 or venous return from tumor CD105 levels had significantly poorer 5-year DSS rate and OS rate.
Choi et al. [75]	2015	Korea	HIF-1α<br>HSP70, -90<br>VEGF<br>IGF-1R<br>P16	IHC	HNSCC	90	NR (30-84)	81/9	-HIF-1α was associated with poor DFS, with borderline statistical significance.<br>-HSP70 expression was correlated with frequent nodal metastasis and thus with poor DFS.
Dalirsani et al. [90]	2020	Iran	EGFR	IHC	OSCC	62	60.7 ±14.77 (33-86)	34/26	-EGFR level showed no correlation with tumor size, location or clinic-pathological<br>characteristics, nor patient survival.
De Aquino et al. [46]	2017	Brazil	VEGF-C<br>VEGFR-3	IHC	Lower lip SCC	50	NR	NR	- Cytoplasmic immunoexpression of VEGFR-3 in the tumor core was associated with metastasis, patient death, and histological grade.
De Oliveira et al. [47]	2013	Brazil	CD31<br>p53	IHC	HNSCC	70	56.4 ± 12.7	62/7	- Patients with locoregional metastasis presented statistically significant higher MVD.
Dos Santos et al. [76]	2012	Brazil	HIF-1α	IHC	OSCC	66	33 cases ≤ 55 yrs<br>33 cases > 55 yrs	56/10	-HIF1a expression can be used as a prognostic marker and predictor of postoperative radiotherapy response, helping the oncologist choose the best treatment for each patient.
De Sousa et al. [48]	2015	Brazil	VEGF-C<br>VEGF-D<br>VEGFR-3	IHC	HNSCC	52	29 cases ≤ 60 yrs<br>23 cases > 60 yrs	43/9	- Although lymphatic spread is a significant event in the progression of HNSCC, the expression of VEGF-C, VEGF-D and VEGFR3 does not correlate with clinicopathological characteristics.
Douglas et al. [77]	2013	UK	HIF-1α<br>CA-IX<br>Bcl-2	IHC	LSCC	382	NR	340/42	-High CA-IX, HIF-1a and Bcl-2 do not add to the prognostic significance of tumor stage and lower hemoglobin in predicting failure of local control.
Dunkel et al. [32]	2016	Finland	HIF-1α<br>CD44	IHC	OSCC	175	65 (median) (23-91)	78/93	-Immunohistochemistry of CD44 and HIF1a may be useful in identification of patients with poor prognoses.

Erkılınç et al. [91]	2022	Turkey	PSMA	IHC	LSCC	51	61±10	50/1	-PSMA associated with poor clinical parameters (i.e. cartilage or local invasion and advanced stage) and there is no correlation with OS, DSS or DFS.<br>-PSMA will be used as a target of alternative or adjuvant treatment options to surgery.
Evans et al. [50]	2019	USA	D240<br>CD31	IHC	HNSCC	200	NR (22-93)	130/70	-Patients with metastatic disease were more likely to have high peritumoral MVD. At multivariable analyses, MVD was not significantly related to DFS and OS.
Franz et al. [92]	2020	Italy	maspin<br>CD105<br>nm23-H1	IHC	LSCC	89	64.2 ± 8.8	82/7	-Seven clusters of LSCCs with different profiles were identified (maspin subcellular location pattern, nuclear nm23-H1 and endothelial CD105 expression).<br>-Two clusters were associated with the best prognosis (Cluster 6 nm23-H1≥10%, non nuclear maspin pattern and endothelial CD105 < 6%) and worst prognosis (Cluster 3 nm23-H1<10%, non-nuclear maspin pattern and endothelial CD105 ≥6%).
Gadbail et al. [51]	2020	India	Ki-67<br>CD105<br>α-SMA	IHC	OSCC	217	NR	NR	-MVD was significantly higher in OSCC compared to oral submucous fibrosis in parameters such as well-differentiated, early TNM stage, non-metastatic, and more than 3-year survival.
Guo et al. [33]	2020	China	IL-1β<br>TGF-β<br>MMP-9<br>angiopoietin-2<br>IF-inducible T-cell α chemoattractant	IHC	Hypo-pharyngeal SCC	60	61.5	60	-IL-1β, TGF-β and MMP-9 may be used as predictors of the effect of induction chemotherapy on poorly differentiated hypopharyngeal cancer.
Guo et al. [93]	2022	China	METTL3<br>m6a<br>CDC25B	q-RT-PCR<br>WB<br>IHC	HNSCC	105	NR	NR	-m6A modification and METTL3 levels are increased in HNSCC, and METTLS3 might be an independent prognostic factor for HNSCC patients.<br>-METTL3 acts as an oncogene to promote cell proliferation, migration, invasion and angiogenesis through the m6A-mediated upregulation of CSC25B.

Haffner et al. [94]	2012	Austria	PSMA<br>COX2<br>FOLH1	IHC	OSCC	96	64 (26-85)	74/22	-PSMA is a strong prognostic marker in OSCC.<br>-COX2 expression and endothelial PSMA levels are associated and could indicate a potential mechanistic interaction between prostaglandin signaling and neo-vascular PSMA expression.
Han et al. [34]	2012	China	CXCR2	IHC<br>qRT-PCR	LSCC	109	60.8 (29-87)	107/2	-Expression of CXCR2 is associated with the development and progression of LSCC. CXCR2 expression may serve as an independent prognostic marker for LSCC patients.
Hong et al. [78]	2013	Australia	HIF-1α	IHC	Oro-pharyngeal SCC	233	58.2 (34–83)	186/47	-The degree of hypoxia as measured by HIF-1a expression does not differ between HPV positive and HPV negative cancers.
Hong et al. [52]	2014	China	Integrin β1<br>VEGF<br>CD105	IHC	Hypo-pharyngeal SCC	50	56.8 (42-79)	47/3	-VEGF overexpression differed significantly across different pathologic grades and different T stages, and regarding N-status.<br>-VEGF expression was positively associated with the MVD.
Ibrahim et al. [95]	2015	Egypt	CD34	IHC	LSCC	80	59.9 ± 8.7 (43-79)	80/0	-Tumors with high MVD are associated with a more aggressive nature, such as nodal metastasis, or recurrence and tumor arising from sites with poorer prognosis (i.e. supraglottis).<br>-No correlation between MVD and recurrence risk in LSCC.
Irani et al. [96]	2018	Iran	CD44<br>(VE)-cadherin<br>Vimentin	IHC	OSCC	63	53.3 (20-70)	40/23	-VE-cadherin, CD44 and Vimentin are related to angiogenesis and VM formation in OSCC, thus in tumor progression and metastasis.
Ishikawa et al. [35]	2014	Japan	IL-33	IHC	Oral Tongue SCC	81	40 cases ≤ 60 yrs<br>41 cases > 60 yrs	50/31	-These data suggest that the IL-33/ST2 axis contributes to tumor aggressiveness and affects the tumor microenvironment.<br>-Immunohistochemical evaluation of IL-33 and ST2 is useful for identifying patients at a high risk for poor prognosis.
Jung et al. [53]	2015	Germany	VEGFA,<br>EFNB2,<br>PECAM1/CD31,<br>ANGPT1, 2	IHC	OSCC	83	63 ± 10.0 (31-92)	57/26	-SCC with lymphatic spread showed higher gene expression rates of VEGFA, EFNB2 and ANGPT2 in moderately differentiated tumors.

Kämmerer et al. [54]	2015	Germany	VEGF	IHC	OSCC	50	60 (31-81)	41/9	-A significantly higher MVD was seen for T3-T4 vs. T1-T2, N+ vs. N0 and G3–G4 vs. G1–G2 OSCCs.<br>-A higher MVD was associated with increased rates of local relapses, more metastases, and a decreased overall and disease-free survival.
Kawasaki et al. [97]	2018	Japan	mTORC1<br>mTORC2	IHC	OSCC	72	26 cases ≥ 67 yrs<br>46 cases < 67 yrs	42/30	-mTORC1 and mTORC2 are overexpressed in OSCC.<br>-mTORC1(–)/mTORC2(+) had significantly low expression of VEGF and HIF1-α.<br>-mTORC1(–)/mTORC2(+) may have a low potential for malignancy in OSCC, and furthermore have correlation with the effectiveness of anti-mTOR drugs.
Kishimoto et al. [79]	2012	Japan	HIF-1α<br>Angiogenin<br>VEGF	ELISA<br>q-RT-PCR<br>WB<br>IHC	OSCC	50	NR	NR	-Angiogenin is up-regulated in the hypoxic environment of oral cancers and its inhibition can have a therapeutic implication.
Ko et al. [55]	2015	Taiwan	VEGF	qRT-PCR	OSCC	60	56 (36-81)	55/5	-The lower mean VEGF mRNA ΔCT value was significantly associated with OSCCs with larger SCC size, neck node metastasis, and more advanced clinical stages.
Koukourakis et al. [56]	2013	Greece	DLL4<br>CD31	IHC	HNSCC	65	NR	NR	-The expression of DLL4 was associated with reduced radio-resistance, presumably by reducing hypoxia and improving chemotherapy accessibility.
Lee et al. [57]	2012	Singapore	HEV	IHC	Oral tongue SCC	65	NR	NR	-The total number of high endothelial venules was found to be significantly associated to disease-free interval and OS. The density of abnormal HEV was significantly higher in patients with lymph nodes metastases.
Li et al. [58]	2013	China	Ang-2<br>VEGFR-3	IHC	OSCC	112	48 cases ≥ 56 yrs<br>64 cases < 56 yrs	81/31	-High Ang-2 expression positively correlated with MVD, and high VEGFR-3 expression positively correlated with lymph node metastasis and lymphatic vessel density.
Li et al. [80]	2013	China	HIF-1α	q-RT-PCR<br>WB<br>IHC	LSCC	86	51 (median) (51-84)	81/5	-HIF-1a is an important regulator for the upregulation of survivin gene expression induced by hypoxia in LSCC cells, and both proteins could be regarded as 2 key predictors of malignant progression and metastasis of LSCC.

Li et al. [98]	2013	China	Integrin v subfamily	IHC	LSCC	64	54 (38-82)	49/15	-expression of integrin αv and β5 subunits were significantly associated with lymphatic metastasis and angiogenesis of LSCC.<br>-αvβ5 might play an important role in invasion and metastases of LSCC.
Li et al. [99]	2019	China	PFKFB3<br>CD163<br>CD31	IHC	OSCC	117	NR	NR	-PFKPB3 levels were positively correlated with CD163 and CD31 expression in OSCC.<br>-PFKFB3 is possibly involved in the angiogenesis by affecting CD163 expression.<br>-Targeting PFKFB3 or blocking CD163+ TAMs infiltration may provide a therapeutic strategy.
Lim et al. [81]	2017	Taiwan	HIF-2α	IHC	OSCC	58	NR	NR	-HIF-2a overexpression can serve as a good biomarker for cancer status for all tumor stages and may predict an early recurrence within two years.
Lin et al. [100]	2012	Taiwan	EPOR	q-RT-PCR<br>WB<br>IHC	OSCC	256	50.9 (26–87)	239/17	-High EPOR expression in OSCC was associated with tumor progression, thus being an important prognostic factor.
Lin et al. [101]	2017	China	α-SMA<br>MMP-9	IHC	OSCC	86	26 cases ≤ 50 yrs<br>60 cases > 50 yrs	56/30	-CAFs plays a role in eliciting angiogenesis and lymphangiogenesis in OSCC, thus promoting progression and metastasis by upregulating MMP-9 and α-SMA expression.
Maqsood et al. [102]	2020	Pakistan	α-SMA<br>CD34	IHC	OSCC	80	45 ± 14.1	50/30	-An increase in the amount of α-SMA positive myofibroblast suggests higher invasive characteristics and weaker prediction of OSCC.
Marioni et al. [59]	2013	Italy	Angiogenin<br>CD105	IHC	LSCC	50	63.4 ± 7.8	41/9	-Univariate analysis showed a significantly higher recurrence rate and shorter DFS in patients with laryngeal SCC with CD105 expression more than 9.0%.<br>-Multivariate analysis found CD105 expression independently prognostic in terms of DFS.

Marioni et al. [103]	2019	Italy	Maspin<br>mTOR	IHC	LSCC	79	64.2 ± 9.1	71/8	-In a subset of LSCC with non-nuclear maspin pattern mTOR expression was significantly higher in patients whose disease recurred, whereas in LSCC with a nuclear msapin pattern, mTOR expression was not significantly associated with prognosis.
Marwah et al. [60]	2016	India	CD105	IHC	HNSCC	50	53.9 ± 12.5<br>(30-82)	36/14	-No significant association was found between MVD, intratumoral-MVD, and peritumoral-MVD and different site of tumor, size of tumor, pushing/infiltrating margin, and different stages of tumors.<br>-Higher MVD, intratumoral-MVD, and peritumoral-MVD was seen in association with lymph node metastasis.
Mendes et al. [83]	2014	Brazil	HIF-1α	IHC	OSCC	56	56 ± 11<br>(34-81)	48/8	-HIF-1α expression in tumor and peritumoral inflammatory cells may play an important role as prognostic tumor marker.
Mermod et al. [19]	2019	Switzerland	CD31	IHC	OSCC<br>Oropharyngeal SCC	56	62.4 ± 12.6	39/17	-Peritumoral CD31 microvascular density was significantly associated with occult lymph node metastasis.
Mishev et al. [105]	2014	Switzerland	MMP-2<br>MMP-7<br>MMP-9<br>MMP-13	IHC	OSCC	61	NR (41-85)	44/17	-MMP-7 and MMP-13 are downregulated in highly invasive and malignant areas of OSCCs.<br>-MMP-7 and MMP-13 are reliable markers of tumor invasion potential, whereas MMP-2 and MMP-9 are not.
Matsui et al. [61]	2015	Japan	VEFG-A,<br>VEGF-C	IHC	Oral tongue SCC	90	65 (median)<br>(19-91)	51/39	-Tumor depth of invasion, pattern of invasion, and VEGF-C expression were associated with the lymph node metastasis of tongue SCC.
Moreno-Galindo et al. [82]	2014	Spain	EGFR,<br>Cyclin D1,<br>p16, p21, p27,<br>p53, p-AKT,<br>HIF-1α<br>Caspase 3, Bcl2	IHC	LSCC<br>Hypo-pharyngeal<br>SCC	51	56 (37-68)	34/7	-Positive expression of p27 and bcl2 had a significant predictive value for chemotherapy response.<br>-Caspase 3, p53, EGFR and p-AKT were not associated with chemotherapy response.
Nair et al. [62]	2015	India	CD105<br>TGF-β1	IHC	OSCC	50	49.92 (25-75)	41/9	- CD105 acted as one of the receptors of TGF-β1 on endothelial cells and induced the angiogenic pathway in OSCC.

Nayak et al. [63]	2013	India	decorin VEGF-A	IHC	Potentially malignant oral lesions, OSCC	180	81 cases < 42 yrs 99 cases ≥ 42 yrs	144/36	- Lymph node metastasis and reduced decorin expression independently correlated with OS in OSCC. -VEGF-A expression had no significant impact on survival outcome.
Ou et al. [84]	2018	China	CA-IX CD34	IHC	HNSCC	100	77 cases < 65 yrs 23 cases ≥ 65 yrs	80/20	-The combination of MVD and CA-IX expression might give additional prognostic information in HNSCC patients with known HPV status.
Pentheroudakis et al. [85]	2012	Greece	VEGF NRP1, 2 HIF-1α	IHC	LSCC	289	63 (median) (36-82)	277/12	-Upregulated mRNA expression of VEGFR1 and VEGFC is associated with poor patient outcome.
Peterle et al. [86]	2018	Brazil	PAI-1 CA-IX VEGF-A	IHC	OSCC	52	64.23 ±13.93	16/36	-PAI-1, CAIX, and VEGFA are potential markers of poor prognosis in OSCC.
Sakata et al. [64]	2019	Japan	HMGA2 VEGF FGF-2	IHC qRT-PCR	OSCC	110	57 (median) (30–87)	66/44	-High expression of HMGA2 was significantly correlated with distant metastasis and poor prognosis. - HMGA2 depletion in OSCC cells reduced the expression of angiogenesis genes.
Schulter et al. [65]	2018	Germany	CD31 VEGF	IHC	LSCC	97	NR	86/11	-A high expression of angiogenic biomarkers was not associated with poor OS in the overall cohort of patients. -High CD31 count was associated with early stage cancer; in this subgroup high VEGF expression correlated with poor survival. -In early stage SCC a high vessel count was associated with an increased recurrence rate.
Shi et al. [66]	2014	China	DKK1 VEGF-C	IHC qRT-PCR WB	LSCC	102	51 cases ≤ 60 yrs 51 cases > 60 yrs	97/5	-High DKK1 expression was significantly associated with T and clinical stage, lymph node metastasis, and tumor size. -Increased DKK1 levels in SCC tissues correlated with elevated VEGF-C and β-catenin.

Siriwardena et al. [87]	2020	Sri Lanka	periostin, HIF-1α, MMP-9, β-catenin, VEGF-C, EGFR	IHC	OSCC	290	NR (31-85)	218/72	POI, level of differentiation, and expression of EGFR are independent prognostic markers for lymph node metastasis.
Soba et al. [37]	2015	Slovenia	p21, p27, p53, cyclin D1 EGFR Ki-67 CD31	IHC	Oro-pharyngeal SCC	59	52 (median) (39-67)	57/2	-Only p27 correlated with survival on multivariate analysis, in addition to the PS of the patients and intensity of the applied therapies.
Starska et al. [107]	2018	Poland	FGFR-1 FGFR-3	IHC WB qRT-PCR	LSCC	137	61.9 ± 8.2 (45-83)	125/12	-FGFR1 and FGFR3 are highly expressed in surgically treated LSCC tissues at both the mRNA and protein levels.<br>-FGFR1 and FGFR3 expression is correlated with LSCC invasion, loco-regional control and clinical outcome.<br>-Expression of FGFR/PI3K/AKT are associated with a more aggressive growth, therefore at risk of early treatment failure and loco-regional recurrence.
Stasikowska-Kanicka et al. [108]	2018	Poland	ADAM10	IHC	OSCC	123	NR (15-75)	NR	-Immunoexpression of ADAM10 in both groups of OLK was significantly decreased in comparison to OSCC groups.<br>-There is a statistically significant correlation between ADAM10 overexpression and the process of angiogenesis.
Supic et al. [67]	2012	Serbia	VEGF-A	RT-PCR	OSCC	114	47 cases < 58 yrs<br>67 cases ≥ 58 yrs	84/30	-VEGF-A -1154 GG genotype could be considered as a prognostic marker of poor survival in advanced-stage OSCC patients.
Swartz et al. [88]	2021	Netherlands	HIF-1α	IHC	HNSCC	941	62.3 ± 10.7	654/287	-High HIF-1a expression is related to poor outcome in oro-pharyngeal SCC and LSCC and better outcome in OSCC; oro-pharyngeal SCC cohort was described in Swartz et al. 2016.

Toyoda et al. [109]	2015	Japan	LAT1<br>CD98<br>CD34<br>p53<br>Ki-67	IHC	Hypopharyngeal SCC	70	68 (median) (37-84)	61/9	-A statistical correlation was recognized between LAT1 and CD98 expression and both expressions were closely associated with tumor cell proliferation.<br>-LAT1 expression was not significantly associated with poor survival.<br>-CD98 expression is an independent prognostic factor for predicting a poor outcome.
Wachters et al. [89]	2020	Netherlands	pAKT, Ki-67, β-Catenin. HIF-1α, CA-IX, OPN, FADD, pFADD, Cyclin D1, Cortactin and EGFR	IHC	LSCC	276	63.5 (median) (33-96)	236/40	-Supraglottic SCCL was associated with higher expression of HIF-1α, Cortactin, EGFR and Ki-67 glottic LSCC demonstrated higher expression of CA-IX and Cyclin D1.
Xia et al. [111]	2014	China	AEG-1	IHC	OSCC	87	60 (34-79)	49/38	-Patients with high AEG-1 expression showed far lower OS rates than those with low expression.<br>-Expression of AEG-1 may be correlated with tumor angiogenesis and metastasis and is a valuable prognostic factor in patients with OSCC.
Yamagata et al. [70]	2017	Japan	CD163<br>CD68<br>CD204	IHC	OSCC	70	NR (28-84)	49/21	-Increased densities of CD68-, CD163- and CD204-positive tumor-associated macrophages were significantly correlated with lymph node metastasis.
Yanagiya et al. [39]	2021	Japan	CXCR7	IHC	OSCC	59	66 (median)	35/24	-High levels of CXCR7 in OSCC blood vessels correlate with adverse disease outcomes, suggesting that CXCR7 is a promising diagnostic and prognostic biomarker.
Yanase et al. [71]	2014	Japan	VEGF-A<br>VEGF-C	IHC	OSCC	61	64.3 (median)	32/29	-VEGF-A expression correlated significantly with lymph node metastasis.<br>-VEGF-C expression was associated with lymph node metastasis, recurrence, and a poorer 5-year survival rate.<br>-VEGF-C was an independent prognostic factor for patients with OSCC.
Zhou et al. [113]	2020	China	nm23-H1<br>CD105	IHC	LSCC	102	68.7 ± 5.4 (60-79)	100/2	-nm23-H1 and CD105 were independent related factors of tumor recurrence.

Abbreviations AEG-1: Astrocyte elevated gene 1, ADAM10: a disintegrin and metalloproteinase-10, α-SMA: α-smooth muscle actin, Ang-2: Angiotensin-2, ANGPT1,2: Angiopoietin 1,2, Bcl-2: B-cell lymphoma 2, CA-IX: carbonic anhydrase-9, CCR7: C-C motif chemokine receptor 7, COX-2: cyclooxygenase-2, CXCR2: chemokine receptor type 2, DLL4: delta like protein, DFS: disease free survival, DKK1: Dickkopf-1, DSS: disease specific survival, EFNB2: Ephrin B2, EGFR: Epidermal growth factor receptor, ELISA: enzyme-linked immunosorbent assay, EPOR: erythropoietin receptor, FADD: FAS-associated death domain protein, FOLH1: folate hydrolase 1, GLUT-1: glucose transporter 1, HEV: High endothelial venules, HIF-1α: hypoxia inducible factor 1α, HMGA2: High-mobility group A protein 2, HNSCC: head and neck squamous cell carcinoma, HSP70,90: heat-shock protein 70,90, IF-inducible T-cell α chemoattractant: interferon-inducible T-cell α chemoattractant, IGF1R: Insulin growth factor 1 receptor, IHC: Immunohistochemistry, IL-1 β: Interleukin 1 β, IL-33: interleukin-33, LAT1: L-type amino acid transporter 1, LOXL-2: Lox-like 2, LSCC: laryngeal squamous cell carcinoma, METTL3: methyltransferase 3, MMP-9: Matrix metalloproteinase-9, mTORC: Mammalian target of rapamycin complex, MVD: microvascular density, NR: not reported, NRP1, NRP2: neuropilin1, 2, OLK: oral leukoplakia, OPN: osteoprotein, OS: overall survival, OSCC: oral squamous cell carcinoma, PAI-1: plasminogen activator inhibitor-1, PECAM-1: Platelet endothelial cell adhesion molecule, PFKFB3: 6-phosphofructo-2-kinase/fructose-2, 6-biphosphatase 3, POI: pattern of invasion, PSMA: prostate-specific membrane antigen, qRT-PCR: quantitative real time protein chain reaction, SEMA3E: semaphorin 3E, SCC: squamous cell carcinoma, ST2: interleukin receptor, TAMs: tumor-associated macrophages, TGF- β: Tissue growth factor- β, VEGF: vascular endothelial growth factor, VM: vasculogenic mimicry, WB: western blot.

Table S3. Quality assessment of the included studies.

	Author	Year	Quality assessment
DIAGNOSTIC MARKERS	Aggarwal et al. [41]	2014	Good
	Aggarwal et al. [31]	2015	Good
	Etemad-Moghadam et al. [49]	2019	Good
	Kayamori et al. [36]	2016	Good
	Mariz et al. [104]	2019	Fair
	Nayak et al. [106]	2015	Good
	Tokmak et al. [38]	2021	Fair
	Troy et al. [110]	2013	Fair
	Uzun et al. [68]	2021	Fair
	Xu et al. [69]	2016	Poor
	Yang et al. [40]	2013	Fair
	Yang et al. [112]	2021	Good
PROGNOSTIC MARKERS	Agena et al. [72]	2021	Good
	Al-Shareef et al. [42]	2016	Good
	Ansari et al. [43]	2020	Good
	Bernstein et al. [73]	2015	Good
	Bertini et al. [44]	2016	Fair
	Bharti et al. [74]	2020	Good
	Chen et al. [45]	2016	Good
	Choi et al. [75]	2015	Fair
	Dalisarni et al. [90]	2020	Fair
	De Aquino et al. [46]	2017	Fair
	De Oliveira et al. [47]	2013	Fair
	Dos Santos et al. [76]	2012	Fair
	De Sousa et al. [48]	2015	Fair
	Douglas et al. [77]	2013	Fair
	Dunkel et al. [32]	2016	Fair
	Erkılınç et al. [91]	2022	Fair

Evans et al. [50]	2019	Good
Franz et al. [92]	2020	Good
Gadbail et al. [51]	2020	Fair
Guo et al. [33]	2020	Poor
Guo et al. [93]	2022	Good
Haffner et al. [94]	2012	Good
Han et al. [34]	2012	Fair
Hong et al. [78]	2013	Fair
Hong et al. [52]	2014	Fair
Ibrahim et al. [95]	2015	Fair
Irani et al. [96]	2018	Fair
Ishikawa et al. [35]	2014	Fair
Jung et al. [53]	2015	Good
Kämmerer et al. [54]	2015	Fair
Kawasaki et al. [97]	2018	Fair
Kishimoto et al. [79]	2012	Poor
Ko et al. [55]	2015	Fair
Koukourakis et al. [56]	2013	Poor
Lee et al. [57]	2012	Fair
Li et al. [58]	2013	Good
Li et al. [80]	2013	Good
Li et al. [98]	2013	Fair
Li et al. [99]	2019	Fair
Lim et al. [81]	2017	Poor
Lin et al. [100]	2012	Good
Lin et al. [101]	2017	Fair
Maqsood et al. [102]	2020	Fair
Marioni et al. [59]	2013	Good
Marioni et al. [103]	2019	Good
Marwah et al. [60]	2016	Fair

Oliveira Mendes et al. [83]	2014	Fair
Mermod et al. [19]	2019	Fair
Mishev et al. [105]	2014	Good
Matsui et al. [61]	2015	Fair
Moreno-Galindo et al. [82]	2014	Fair
Nair et al. [62]	2016	Good
Nayak et al. [63]	2013	Good
Ou et al. [84]	2018	Fair
Pentheroudakis et al. [85]	2012	Fair
Peterle et al. [86]	2018	Good
Sakata et al. [64]	2019	Fair
Schulter et al. [65]	2018	Fair
Shi et al. [66]	2014	Fair
Siriwardena et al. [87]	2020	Good
Soba et al. [37]	2015	Poor
Starska et al. [107]	2018	Good
Stasikowska-Kanicka et al. [108]	2018	Fair
Supic et al. [67]	2012	Good
Swartz et al. [88]	2021	Good
Toyoda et al. [109]	2015	Good
Wachters et al. [89]	2020	Fair
Xia et al. [111]	2014	Fair
Yamagata et al. [70]	2017	Fair
Yanagiya et al. [39]	2021	Good
Yanase et al. [71]	2014	Fair
Zhou et al. [113]	2020	Fair
